# Impact of limited sample size and follow-up on single event survival extrapolation for health technology assessment: a simulation study

**DOI:** 10.1186/s12874-021-01468-7

**Published:** 2021-12-18

**Authors:** Jaclyn M. Beca, Kelvin K. W. Chan, David M. J. Naimark, Petros Pechlivanoglou

**Affiliations:** 1 Institute of Health Policy, Management and Evaluation, University of Toronto, Toronto, Canada; 2 Ontario Health (Cancer Care Ontario), Toronto, Canada; 3 Canadian Centre for Applied Research in Cancer Control (ARCC), Toronto, Canada; 4 Sunnybrook Health Sciences Centre, Toronto, Canada; 5 Child Health and Evaluative Sciences, Hospital for Sick Children, Toronto, Canada

**Keywords:** Survival, Extrapolation, Health technology assessment, Economic evaluation, Decision modelling, Simulation

## Abstract

**Introduction:**

Extrapolation of time-to-event data from clinical trials is commonly used in decision models for health technology assessment (HTA). The objective of this study was to assess performance of standard parametric survival analysis techniques for extrapolation of time-to-event data for a single event from clinical trials with limited data due to small samples or short follow-up.

**Methods:**

Simulated populations with 50,000 individuals were generated with an exponential hazard rate for the event of interest. A scenario consisted of 5000 repetitions with six sample size groups (30–500 patients) artificially censored after every 10% of events observed. Goodness-of-fit statistics (AIC, BIC) were used to determine the best-fitting among standard parametric distributions (exponential, Weibull, log-normal, log-logistic, generalized gamma, Gompertz). Median survival, one-year survival probability, time horizon (1% survival time, or 99th percentile of survival distribution) and restricted mean survival time (RMST) were compared to population values to assess coverage and error (e.g., mean absolute percentage error).

**Results:**

The true exponential distribution was correctly identified using goodness-of-fit according to BIC more frequently compared to AIC (average 92% vs 68%). Under-coverage and large errors were observed for all outcomes when distributions were specified by AIC and for time horizon and RMST with BIC. Error in point estimates were found to be strongly associated with sample size and completeness of follow-up. Small samples produced larger average error, even with complete follow-up, than large samples with short follow-up. Correctly specifying the event distribution reduced magnitude of error in larger samples but not in smaller samples.

**Conclusions:**

Limited clinical data from small samples, or short follow-up of large samples, produce large error in estimates relevant to HTA regardless of whether the correct distribution is specified. The associated uncertainty in estimated parameters may not capture the true population values. Decision models that base lifetime time horizon on the model’s extrapolated output are not likely to reliably estimate mean survival or its uncertainty. For data with an exponential event distribution, BIC more reliably identified the true distribution than AIC. These findings have important implications for health decision modelling and HTA of novel therapies seeking approval with limited evidence.

**Supplementary Information:**

The online version contains supplementary material available at 10.1186/s12874-021-01468-7.

## Introduction

Health decision models play an important role in health technology assessment (HTA) and reimbursement decision-making for new drugs and technologies [1–3]. Health decision models are a systematic approach to synthesizing information regarding relative costs and outcomes associated with alternative options [4]. Health decision models aim to estimate mean survival with various strategies and are informed by clinical data to determine health state occupancy time and associated transition risks [3–5]. Parametric survival analysis methods are used to model event hazards from clinical time-to-event data and extrapolate to lifetime horizons to estimate mean survival for decision modeling [6–9]. Given the large effect extrapolation choices may have on decision model results, emphasis has been placed on robust, systematic approaches for extrapolation choices as best practice [1, 8, 10, 11].

Oncology is an area of rapid clinical development [12]. Given the number of experimental trials underway and specific eligibility criteria applied, clinical trials in oncology often involve small samples [12]. Of all investigational trials registered and active on clinicaltrials.gov as of April 2021, 24% are investigating pharmaceuticals for oncology, among which, 69% of phase II and 24% of phase III trials plan to enroll fewer than 100 patients per arm [13]. In reviews of recent FDA approvals, the majority of trials for oncology indications had less than 200 participants [14], and oncology was more likely than other disease areas to obtain regulatory approvals based on surrogate endpoints, single-arm evidence, and a single pivotal trial [15–18]. There is also growing use of innovative and adaptive trial designs to address personalized medicine. It is evident that regulatory and HTA agencies are becoming increasingly reliant on studies with limited data to inform clinical and economic assessments of new therapies [17–20].

Time-to-event data from clinical trials are affected by the number of patients enrolled, how long patients are followed, and rate of the event of interest [21]. While incidence and event rate are epidemiological characteristics, the number of sites involved to enroll patients, total target sample size and duration of follow-up are controlled by researchers designing the study. When samples are small or follow-up is short, there are limited data with which to fit parametric distributions for extrapolation. Currently, we do not have a comprehensive quantitative understanding of the impact sample size and follow-up characteristics of clinical studies may have on parametric extrapolation, and resulting impact on decision models [21].

The objective of this study was to assess performance of standard commonly-used parametric survival analysis techniques for extrapolation of time-to-event data from clinical trials under conditions of limited data due to small samples or short follow-up. We assess performance by quantifying coverage and error of estimates for survival outcomes from parametric extrapolations of simulated datasets.

## Methods

### Approach

A simulation was designed to assess coverage and error when extrapolating survival data derived from a known data-generating process under various conditions with limited data.

We evaluated different levels of sample size, *n*_*obs*_, by randomly selecting study samples from a simulated population of individuals with complete observations (i.e., all death times known). We then evaluated different durations of follow-up, by artificially censoring the complete data for each sample after specific proportions of patients had events (proportion of events, *p*_*e*_), thereby controlling the degree of censoring (1 – *p*_*e*_). We used this approach to evaluate different levels of follow-up in a consistent and controlled manner across all datasets.

We simulated patient populations for four scenarios to examine two levels of hazard (event rate, λ), and accrual period. Accrual was used to mimic clinical trial enrollment with simulated individuals entering the trial at random start times during the accrual period, thereby staggering observation times.

In the primary scenario, accrual was conducted over 9 months, and the event rate was selected in a way that would produce a median event time of approximately 9 months. These were considered “short” accrual and “high” event rate. Three additional scenarios were conducted and presented in Supplemental Files, to ensure that results were not driven by choice of values for these parameters. The scenarios used longer accrual, lower event rate, and both longer accrual and lower event rate combined.

### Simulation setup

#### Data generation approach

Data were generated using a stochastic process [22, 23]. To simulate a trial with staggered enrollment, randomly generated study enrollment times *t*_1*k*_ for each individual were sampled from a uniform distribution between one and maximum accrual time, *T*_1_ (9 months for Scenarios 1, 3; 30 months for Scenarios 2, 4). This approach of generating random enrollment times is similar to previously proposed methods of simulation of clinical trial data with survival endpoints [21, 24]. Event times in days for each individual, *t*_2*k*_, from study enrollment were sampled from an exponential distribution with constant rate, *λ*_12_ = 0.0025, to estimate median survival of approximately 9 months (− *log* (0.5)/0.0025 = 277 days) (Scenarios 1, 2). The study was repeated with a lower event rate, *λ*_12_ = 0.00075, (− *log* (0.5)/0.00075 = 924 days, approximately 30 months) (Scenarios 3, 4).

#### Simulated populations and datasets

We simulated *k =* 50,000 individuals for each scenario from the data generating process to form populations. We chose six levels of sample size, *n*_*obs*_ = {30, 60, 90, 120, 250, 500}, and randomly selected study samples from the simulated population for each of *i* = {1, …, *n*_*sim*_ = 5,000} repetitions. We then artificially censored each sample dataset to analyze the data at various levels of follow-up. The complete data from each sample was used to identify the follow-up times needed (from the start of the study) to observe different proportions of events. These times were then used to create multiple artificially censored versions of each sample in order to imitate different levels of follow-up for that sample. For example, when 10% of patients had experienced the event (3 patients out of 30 in the smallest sample size group), the sample’s remaining follow-up was artificially truncated and all remaining accrued patients were censored in order to form the dataset to analyze the shortest level of follow-up. To form a dataset for the next level of follow-up, this process was repeated by artificially censoring the complete patient data for the sample at the time from the start of the study when 20% of patients experience the event. Since patients accrued to the study over time from the study initiation date, patients have different lengths of follow-up from time of enrollment to when a study is stopped and administratively censored on a specific calendar day. This allowed us to evaluate the impact of changing the length of follow-up in that sample, in a manner similar to administratively censoring a clinical trial at a given time after targeted number of events are observed. The approach also allowed evaluation in a consistent manner across samples for different proportions, including complete follow-up (all events observed). Within each repetition and sample size, artificially censored datasets were created based on deciles of proportions of events, *p*_*e*_ = {10%, 20%, …, 100%}, creating ten levels of follow-up. Thus, we included *n*_*sim*_ = 5,000 repetitions, where within each repetition there were six levels of *n*_*obs*_, which in turn were analyzed after every 10% increase in number of events observed, for 10 levels of *p*_*e*_, producing a total of 300,000 “datasets”. We refer to each combination of sample size and level of events observed as a “grouping”.

After datasets were set up from start of the study to reflect staggered observation times, clock was reset at enrollment for survival analysis of the endpoint of interest. R statistical software (v 4.0.3) was used to simulate and analyze data, using the *gems* and *flexsurv* packages (with default parameterizations), respectively [25]. See Supplemental File 1 for more details of simulation methods and data setup and Supplemental File 2 for more details of simulated populations and datasets.

#### Simulation plan

The study was designed according to the aims, data generating mechanism, estimands, methods, and performance measures (ADEMP) guidelines for simulation studies (Table 1) [26].

**Table 1:** Simulation plan according to ADEMP guidelines

Category	Description
Aims	The aim of this study was to assess the performance of standard parametric survival analysis techniques for analysis of time-to-event data from clinical trials under conditions of limited data due to small samples or short follow-up.
Data generating mechanism	Data were generated for the event of interest from an exponential survival distribution, characterized by a constant hazard rate, *λ*.
Estimands and population targets	- Exponential distribution of event times - Median survival time, *t where S*(*t*) = 0.5 - One-year landmark survival probability, *S*(*t*) *where t* = 365 *days* - Population time horizon, *TH*_*pop*,_ defined at 1% survival time, *t where S*(*t*) = 0.01 - Restricted mean survival time (RMST) estimated at time horizon *TH*_*pop*_
Methods	Simulated populations were created and *n*_*sim*_ = 5000 repetitions drawn. Each repetition included six levels of sample size, *n*_*obs*_ = {30, 60, 90, 120, 250, 500}. Within each repetition and sample size, artificially censored datasets were created based on deciles of proportions of events, *p*_*e*_ = {10%, 20%, …, 100%}, creating ten levels of follow-up. Standard parametric distributions (exponential, Weibull, log-normal, log-logistic, generalized gamma and Gompertz) were fitted to each grouping for each repetition, nonconverging or implausible fits removed, and estimated model parameters (estimators) collected from extrapolated survival curves: - Information criteria (IC) to determine the best-fitting distribution - Median survival time, *t where S*(*t*) = 0.5 - One-year landmark survival probability, *S*(*t*) *where t* = 365 *days* - Sample time horizon *TH*_*i*_, (1% survival time), *t where S*(*t*) = 0.01 - Population time horizon RMST (RMST estimated at *TH*_*pop*_) - Sample time horizon RMST (RMST estimated at *TH*_*i*_)
Performance measures	- Proportion identifying the true distribution as best fitting - Coverage - Error ◦ Mean absolute error (MAE) ◦ Mean absolute percentage error (MAPE) ◦ Root mean squared error (RMSE) ◦ Probability of 20% error

#### Estimands and population targets

An estimand is the true population quantity that the simulation will target [26]. Using a known distribution for generating simulated data allowed us to evaluate outcomes from samples against the true population parameters. In addition to an exponential distribution of event times, quantities of interest from the population fitted model included: median survival time; one-year survival probability; an estimated “population lifetime” time horizon, *TH*_*pop*_, which we defined as the time at which the extrapolated curve from the fitted survival distribution reached 1%, (i.e., 99th percentile of the survival distribution); and restricted mean survival time (RMST) for the population estimated at the population lifetime time horizon *TH*_*pop*_. Population estimands are presented in Supplemental File 2 (Table S2–1).

#### Analytic methods

We fitted standard parametric distributions (exponential, Weibull, log-normal, log-logistic, generalized gamma and Gompertz) to the time-to-event datasets for each replication and grouping to project the survival curves beyond the length of follow-up of the “observed”, artificially censored datasets. We removed any fitted model that failed to converge or met prespecified conditions that would render a fitted survival model as implausible. Conditions that were considered implausible included: failed to produce survival probabilities descending after time 0, produced wide 95% confidence intervals (CI) that spanned > 80% probability of survival at the first event time, infinite values for estimators and associated CIs, or median survival times beyond the maximum event time in the simulated population.

Despite our knowledge of the true distribution, the more practical application is dependent on the performance of the extrapolation when the true distribution is not known. There are multiple mechanisms by which the best-fitting distribution is chosen for extrapolation in practice. We present the outcomes from the known or best-fitting distribution according to two most commonly used statistical criteria. Goodness-of-fit information criteria (IC) statistics (Akaike information criterion [AIC] and Bayesian information criterion [BIC]) were calculated from all remaining models to identify the best-fitting distribution for each dataset. IC corrected for sample size (AICc and BICc) were also explored, although current guidance and most statistical packages present only uncorrected AIC and BIC [8].

The following estimated model parameters (estimators) were extracted for each target estimand from the extrapolations of the fitted exponential model and best-fitting distribution according to each IC: median survival time; one-year survival probability; sample “model-estimated” lifetime time horizon, *TH*_*i*_, which was the time at which the extrapolated curve from the fitted survival distribution reached 1% (or 99th percentile of the survival distribution); and RMST estimated at two different times: population time horizon *TH*_*pop*_, and sample’s model-estimated time horizon *TH*_*i*_. RMST is equivalent to an economic decision model’s estimate of survival (life-years) assessed over a given time horizon. The population lifetime time horizon, *TH*_*pop*_, was used to estimate RMST at a common time across all groupings and repetitions. The sample “model-estimated” lifetime time horizon, *TH*_*i*_, was also used to estimate RMST in order to replicate a modelling approach of determining the time horizon and life-years from the modelling output (i.e., run the model for a time horizon until nearly all patients have died), which might be used when the population’s true time horizon is unknown.

#### Performance measures

Several measures were used to evaluate performance in targeting the population quantities of interest.

*The proportion of repetitions identifying the true distribution as best fitting* assessed groupings where IC from an exponential survival model was lowest among the fitted distributions.

*Coverage* and *error* were assessed with the distribution correctly specified as exponential and from best-fitting distributions selected by each type of IC. *Coverage* assessed the proportion of repetitions with CIs containing the true population quantity. To examine *error,* we assessed *mean absolute percentage error (MAPE),* as well as *mean absolute error (MAE)*, and *root mean squared error (RMSE)*, calculating average error between the estimate from each repetition and the true population fitted quantity. As these three measures provided similar information and qualitative interpretation regarding average magnitude of error, we focused on MAPE for ease of interpretation in the main results and presented MAE and RMSE for the primary scenario in Supplemental File 3 (S3). We also defined another measure, *probability of 20% error*, to assess the proportion of repetitions that produced estimates with an absolute value > 20% of true population fitted quantity in each grouping, as an estimate of the chance a trial produced a potentially meaningful magnitude of difference [27, 28].

Coverage and error for each estimand were calculated for each grouping with respective Monte Carlo standard errors. Formulas for each performance measure are included in Supplemental File 1.

The approach was repeated for all four scenarios to assess varying accrual and event hazard rates; results presented in Supplemental File 4 (S4). The results for all four scenarios are also available in an interactive tool for ease of interpretation (https://survsim.shinyapps.io/survsim).

## Results

### Nonconverging or implausible fits

Fitting to datasets with very small samples or short follow-up were more likely to result in survival models that did not converge or produced implausible results (Fig. S2-3). Among the 1.8 M distributions fitted (300,000 datasets × 6 distribution types), fewer than 10% were defined as nonconvergent or with implausible fit. However, among simulations with shortest follow-up, nearly 50% of fitted models for the smallest sample size and approximately 20% for the largest sample size were associated with one or more of the conditions. The highest incidence of nonconvergence or implausible fits occurred in fitting the generalized gamma model, followed by the Gompertz model. All repetitions had at least one survival distribution converge and/or be considered plausible according to the prespecified conditions for each grouping; thus, no repetition’s grouping had to be removed from the simulation.

### Proportion of repetitions identifying the true distribution as best-fitting

**Fig. 1:**
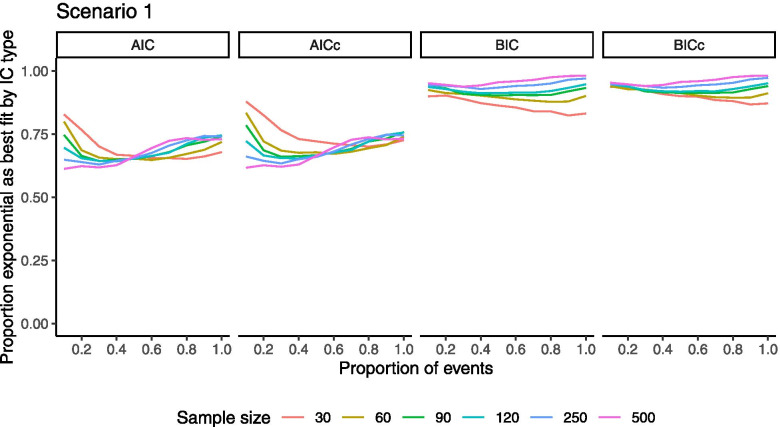
Proportion of repetitions identifying exponential distribution as best-fitting according to AIC or BIC, scenario 1 AIC = Akaike information criteria, BIC = Bayesian information criteria, c = corrected

Generally, the true distribution was more likely to be correctly identified using statistical goodness-of-fit IC with larger samples and more observed events (Fig. 1, findings held across scenarios, Fig. S2-4). Better identification of the true exponential distribution was observed using BIC compared to AIC. With AIC, approximately 70–80% of the repetitions identified the true exponential distribution as best-fitting, even with complete follow-up. With BIC, the true exponential distribution was more commonly identified with larger sample size, and among larger samples, the true exponential distribution more commonly identified with longer follow-up. With the largest sample size, BIC identified the true exponential distribution as best-fitting in nearly 98% of repetitions. With small samples, there was no improvement with longer follow-up using either IC.

IC corrected for small samples (AICc and BICc) improved identification of the true exponential distribution among the three sample size groups below 100, though the improvement was marginal (5% or less across groupings); as expected, larger sample size groups were unaffected.

### Coverage

**Fig. 2:**
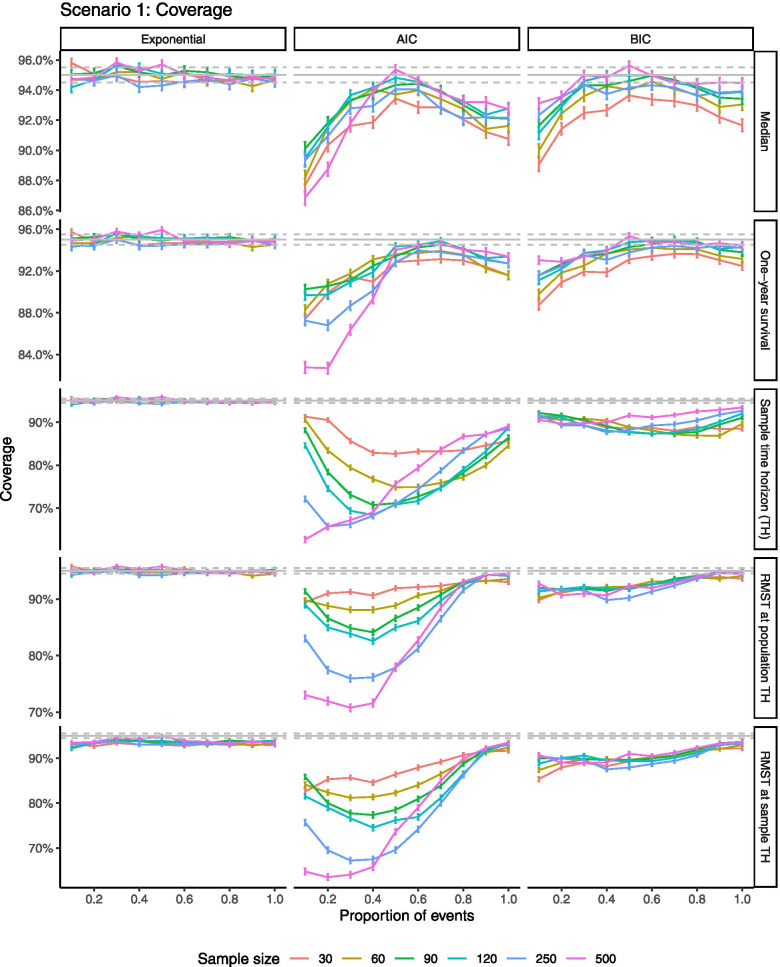
Coverage when distribution is correctly specified as exponential and when chosen by AIC/BIC AIC = Akaike information criteria, BIC = Bayesian information criteria

When the distribution was correctly specified, median survival time, one-year survival probability and RMST estimated at the fixed population time horizon (*TH*_*pop*_), approximated nominal coverage, i.e., 95% of repetition CIs contained the true estimand (Fig. 2, left panel). Using a time horizon based on the individual sample’s 1% survival probability, *T*_2,*i*_, sample RMST CIs contained the true RMST slightly less than 95% of the time (under-coverage).

When the true distribution was unknown and best-fitting curves selected by IC, less than 95% of repetition CIs contained the true estimand across all sample sizes (Fig. 2, middle and right panels). Estimates from best-fitting curves identified by BIC produced better coverage relative to AIC and approached nominal coverage with longer follow-up. For AIC, patterns differed by sample size; with short follow-up, larger samples produced larger deviations from nominal 95% coverage than small samples (due to much wider CIs associated with small samples), but with longer follow-up better approximated nominal coverage than small samples. In estimating median survival, after approximately 50% of events coverage declined with longer follow-up for all sample sizes, suggesting increased precision of narrower CI around a biased value. Selection of distributions with corrected ICs did not affect the results relative to uncorrected ICs (Fig. S3-1). Similar results were observed across scenarios (Fig. S4-1:3).

### Error

**Fig. 3:**
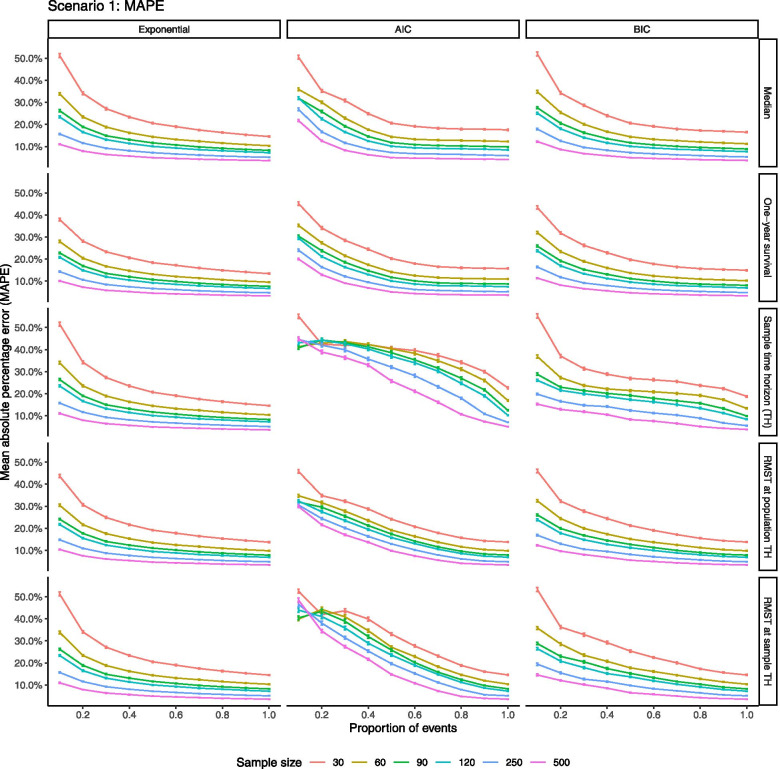
Mean absolute percentage error (MAPE) when distribution correctly specified and when chosen by AIC/BIC AIC = Akaike information criteria, BIC = Bayesian information criteria

There was clear and consistent reduction in error with increasing proportion of events observed and with increased sample size for all outcomes assessed. Given consistency, MAPE was presented for ease of interpretation; MAE and RMSE followed similar patterns (Fig. 3, Fig. S3-2:3). Error was more markedly reduced by larger samples than longer follow-up; complete follow-up of a small sample produced larger error than limited events in a larger sample. When the true distribution was correctly specified, all outcomes demonstrated similar MAPE. Samples of 30 patients demonstrated an average 50% difference from true values with short follow-up and 15% difference with full follow-up, while samples of 500 patients produced much smaller error of approximately 10% after short follow-up and reduced further with additional events.

Error was similarly large at small sample sizes and short follow-up regardless of whether the distribution was correctly specified, with the shortest follow-up for 30 patients exhibiting MAPE of approximately 50% in all cases. On the other hand, error from larger samples after short follow-up was much larger when the distribution was selected by IC than when correctly specified, particularly with AIC. Moreover, reduction in error with longer follow-up was much more gradual for sample time horizon and RMST estimates, when distributions were chosen by IC, particularly AIC. For example, more than 40–60% of events had to be observed in larger samples to achieve comparable MAPE at 10% of events when the true distribution was correctly specified. In another framing, if one was willing to accept a risk of 10–20% in estimates, sample sizes of 250 or more would be likely to suffice regardless of follow-up time as long as BIC were used for model selection. However, to achieve similar precision in smaller samples, at least 50% of events would need to have been observed, and possibly higher for reliable estimation of time horizon and RMST. As with other results, correction for small samples did not improve performance relative to uncorrected IC (Fig. S3-4).

**Fig. 4:**
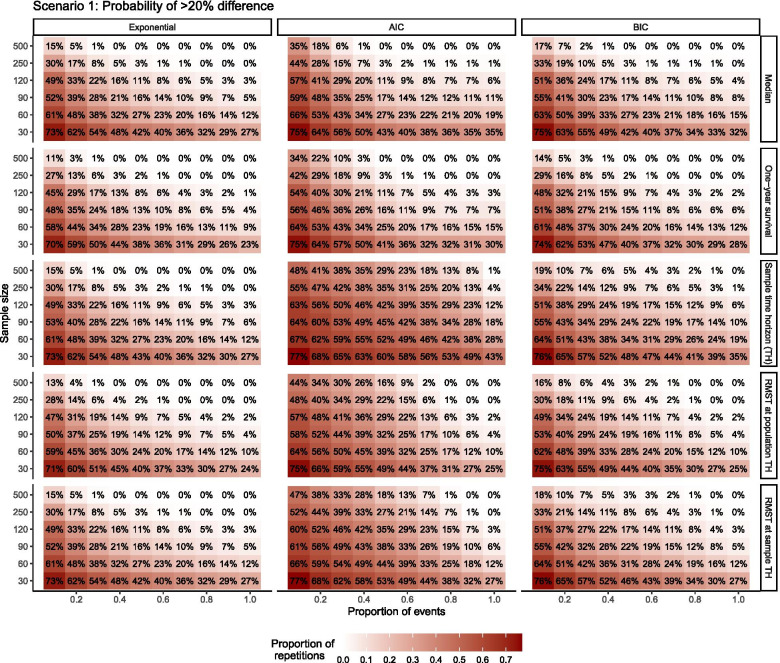
Probability > 20% difference from population value when distribution correctly specified and when chosen by AIC/BIC AIC = Akaike information criteria, BIC = Bayesian information criteria

To further explore error, the probability that a single trial produced a large magnitude of error (> 20%) was also examined in a heat map as a guide for interpreting findings of trials with limited data (Fig. 4). Most repetitions produced large differences compared to true values when samples were small. Over 70% of repetitions produced results with greater than 20% difference from true population values for all outcomes when few events were observed, and more than 25% produced such differences with complete follow-up, regardless of whether the distribution was correctly specified. Nearly all estimates from samples of 500 patients fell within 20% of true values with less than 40% of events observed when the distribution was correctly specified or selected using BIC. Specifying the distribution with AIC produced larger probability of > 20% error across all outcomes in all but the best groupings (full follow-up of largest samples), particularly for sample time horizon and RMST.

The magnitude of average error and probability of > 20% error in an individual repetition were remarkably similar for nearly all outcomes across scenarios (Fig. S4-4:9).

## Discussion

This study provides findings concerning the validity of extrapolations of limited data to populate health decision models, as studies with small samples may risk large error in estimates relevant to HTA. Key findings are summarized in Table 2. Error in point estimates from a sample were found to be strongly associated with sample size and completeness of follow-up. This error existed even when the event distribution was correctly specified for small samples, while correctly specifying the event distribution reduced magnitude of error in larger samples.

**Table 2:** Summary of key findings

• There is a large risk of error when extrapolating clinical time-to-event data with small sample sizes, which is observed regardless of whether the underlying event distribution has been correctly specified when undertaking extrapolation. Error is more markedly reduced by larger samples than by observing more events with longer follow-up alone. • Uncertainty may not be sufficiently captured within estimated confidence intervals when extrapolating limited clinical data for use in decision models, suggesting that probabilistic analysis is not sufficient to overcome the limitations of small samples or of short follow-up in large samples. • Identifying lifetime time horizon based on the model’s extrapolated output will not reliably estimate mean lifetime survival and its uncertainty. • For data with an exponential event distribution, AIC less frequently correctly identified the true distribution and performed very poorly in estimating outcomes and appropriately capturing their uncertainty compared to selections based on BIC.	

When the true distribution was correctly specified to extrapolate limited data, population values were likely to be captured reasonably well within a sample’s CI, regardless of sample size or follow-up. However, good coverage from limited data is obtained from wide CIs, which means a large degree of uncertainty. In the more practical context in which the distribution is not known, an estimate’s CI could not reliably be expected to include the true estimate. Longer follow-up alone did not necessarily improve precision of estimates of median survival or one-year survival probability, as longer follow-up may only produce more confidence in a biased estimate. Using a time horizon driven by the sample’s extrapolated curve was associated with under-coverage regardless of the distribution selection method. These findings suggest establishing a model’s time horizon based on the extrapolated output will not reliably estimate mean lifetime survival and its CI, and probabilistic analysis is not sufficient to overcome the limitations of small samples or of short follow-up in large samples.

When evaluating data with an exponential event distribution, AIC performed very poorly in estimating parameters and their uncertainty. BIC correctly identified the true distribution more frequently than AIC, particularly with larger samples and longer follow-up, though longer follow-up provided limited improvement among small samples. Moreover, selection with BIC produced better coverage and reduced error relative to AIC. It is evident the mechanism by which the best-fitting distribution is chosen affects coverage and error of the estimates, with the key driver of the difference between these being accurate characterization of the hazard. With a true exponential distribution, we also found IC corrections for small samples slightly improved the identification of the true distribution but did not appreciably improve coverage or reduce error. IC corrections are more theoretically appropriate with small samples and converge to their uncorrected counterparts with larger samples [29], but given their limited use, it is reassuring to note that differences could be minor in practice.

Pronounced differences were not observed across scenarios comparing relationships between accrual time and event time, suggesting the findings are not primarily driven by these factors, but rather the relationship with follow-up as a proportion of events regardless of the speed at which these events occur.

There is very limited guidance to inform the appropriateness of using a given sample size or completeness of follow-up for evaluating time-to-event measures for economic evaluation decision models. In planning a clinical trial, sample size and analysis timing are determined by the primary outcome. Randomized phase III oncology studies base sample size on anticipated average effect size of the intervention relative to controls on time to progression or death, accounting for accrual and potential attrition [30]. Phase II oncology trials may only assess an intermediate endpoint such as tumour response, comparing the single treatment arm outcomes with historical controls [31]. Response is typically assessed early in treatment, resulting in limited sample sizes and follow-up for exploratory time-to-event endpoints and no formal statistical criteria informing the time-to-event evaluation [15]. While a phase II trial is not intended to determine treatment efficacy, there is growing precedent for such trials to inform regulatory and HTA decisions, with exploratory time-to-event outcomes forming the basis of clinical and economic assessments [15]. A recent review of Canadian oncology drug review demonstrated that about one quarter of submissions in the last decade were made on the basis of an early-phase clinical trial with surrogate endpoints only [20]. Innovative trial designs are also becoming more commonplace in oncology, including master protocol designs or basket trials for very rare conditions based on molecular alteration rather than histology (tissue type/site). These studies are designed to inform regulatory decision-making as opposed to HTA [12, 15, 32, 33]. In the era of precision medicine, such designs may be more efficient and flexible for drug development, but pose challenges for appraisal given they are often early-phase, nonrandomized, and involve extremely small samples with potentially heterogeneous clinical subtypes and treatment effects [32, 34]. Yet, regulatory approvals have been granted for therapies studied with such trials, creating challenges for economic evaluation decision modelling and HTA [34, 35]. Our study findings raise questions regarding the use of survival data derived from small, earlier-phase trials and those reporting interim analyses and secondary time-to-event outcomes. It raises considerable concerns for using limited clinical data in decision models, given the risk of under-coverage and large error for the estimation of time horizon and RMST. In circumstances of single arm, non-comparative data, it would be difficult to make any inference based on naïve or unanchored comparisons of absolute survival outcomes given the high risk of error associated with a single trial, especially with small, highly censored samples, despite common use of this approach evaluating phase II trial data against historical controls [17, 19, 20, 36, 37].

No study has examined in depth the relationship between sample size, completeness of follow-up, and performance of extrapolation methods for estimation of clinical and economic decision-modelling parameters. Aspects of this study have been evaluated previously, including impact of accrual and follow-up on estimation of relative and non-constant treatment effects [21], case studies on the impact of survival distribution choice on estimates of extrapolated hazard and mean survival [11], and performance of IC and bias in RMST estimates in simulated results from clinical trial case study scenarios [38]. Our simulation study design analyzes several main factors affecting time-to-event outcomes across a full range of sample size and follow-up, across different accrual and event rates, and examines multiple outcomes relevant to HTA.

Our study had several limitations. Firstly, the exponential distribution assumes a constant hazard rate over time and may not be generalizable to other contexts. A simulated dataset generated with exponential distribution was chosen to control the data-generating mechanism and limit additional “noise” from a time-varying hazard. However, disease processes commonly produce non-constant hazards, which could alter the study dynamics. Identification of the exponential distribution as best fitting in a larger proportion of simulations with BIC than AIC is not unexpected given that a larger penalty is incorporated into the BIC formula for number of model parameters, thus favouring more parsimonious parametric distributions. However, another recent study that simulated data from several clinical trials also found better performance with BIC, despite non-constant hazards in the case studies used [38]. Thus, we expect the results will hold in settings with non-constant hazards. However, it is not known whether these findings are due to the simulation study designs; future studies are needed to assess generalizability. Moreover, the added benefits of characterizing survival with RMST in the setting of non-constant (and non-proportional, when comparing two treatment) hazards as opposed to traditional estimates (e.g., median) are not appreciated in this study context. However, RMST is equivalent to estimating life-years in economic decision modelling and thus, the potential for added uncertainty in the magnitude of error and coverage for RMST relative to medians even in the context of constant hazards is an important finding. Additionally, outside of non-converging or illogical model estimation, best-fitting curves were selected based on IC alone, which simplifies selection in practice that typically includes visual inspection and validation against external data or opinion, where possible. However, overreliance on fit statistics has been observed in reviews of extrapolation approaches in HTA [9, 10]. Though removal of failed or implausible results appeared to improve selection of the true distribution slightly (via process of elimination), this seemed limited to short follow-up as a minimal measure only. Further planned studies will aim to evaluate the robustness of the findings across a larger range of scenarios that include non-constant hazards, multiple events, and hazards not derived from a standard parametric distribution. Lastly, we based follow-up time according to proportion of events observed, with the lowest proportions being observed prior to full accrual in some instances. Though analyses can be conducted prior to full accrual in event-driven designs [39], in many trials, analysis would not proceed until a more substantial number of events had occurred or after full target accrual. However, the approach allowed a full assessment of the range of events across all repetitions. Moreover, findings may be relevant to longer-term secondary outcomes such as overall survival, when analysis following low proportions of events may be especially likely.

## Conclusion

In conclusion, this study found that when the true data generating mechanism is based on an exponential distribution, BIC more commonly correctly identified the true distribution than AIC. Limited clinical data in the form of small samples or short follow-up of large samples are at risk of producing large error in estimates relevant to clinical and economic assessment used in HTA regardless of whether the correct distribution is specified, and the associated uncertainty in the estimated parameters may not capture the true population values.

## Supplementary Information

**Additional file 1.** Additional details for simulation study design.

**Additional file 2.** Additional details for simulated populations and datasets.

**Additional file 3.** Additional results for Scenario 1.

**Additional file 4.** Additional results comapring across all four scenarios.

## Data Availability

The datasets generated during and analyzed during the current study are available in the authors’ GitHub repository. https://github.com/jaclynbeca/extrapolation
