# Supplementary material for: Impact of limited sample size and follow-up on single event survival extrapolation for health technology assessment: a simulation study
Source: BMC Med Res Methodol. 2021 Dec 18;21:282. doi: 10.1186/s12874-021-01468-7 (PMC8684239; doi:10.1186/s12874-021-01468-7)
Supplement: Supplementary file 1 — Additional file 1. Additional details for simulation study design. [file 12874_2021_1468_MOESM1_ESM.docx]

# Supplemental File 1: Additional details for simulation study design

Table of Contents

[Supplemental File 1: Additional details for simulation study design 1](#_Toc81819307)

[Framework 2](#_Toc81819308)

[Details of sampling methods 3](#_Toc81819309)

[Formulas for performance measures 7](#_Toc81819310)

## Framework

A multistate process is a stochastic process, $Y\left( t \right), t \epsilon\left[ 0, \infty\right),$ with a finite number of *S* states [24]. The process has an initial distribution, $\Pr\left( Y\left( 0 \right)=j \right), j \epsilon\{1,\ldots S)$, as each sampled patient population begins in one of the $S$ states [25]. In a time-homogeneous (Markov) multistate process, each patient moves from an initial distribution, irrespective of time in the current or in previous states. The process is characterized by transition intensities, or instantaneous rates of transition between states from $j$ to $k$ at time $t$,

$\lambda_{j,k}\left( t \right)= \lim_{\delta t\to0} \frac{P\left( Y\left( t+\delta t \right)=k \right|Y\left( t \right)=j)}{\delta t}$.

Transition intensities can be arranged into a matrix illustrating the transitions from each state to the other, known as a transition intensity matrix (TIM), to fully characterize the process [24]. Univariate time-to-event data (traditional survival analysis) can be conceived as originating from a simple multistate process, with two states and one transition between them that defines the time of the event (e.g., death). The transition intensity for such a multistate process is known as a hazard rate, the instantaneous rate of experiencing the event, conditional on being event-free (i.e., in the initial state) at that time.

## Details of sampling methods

A stochastic process using a multistate framework was used for the data generation process to create individual-level data for enrollment and event times. A three-state multistate model with two transitions was established to simulate the population for each scenario. The simulated population included *k =* 50,000 individuals, who were subject to two forward transitions (see Figure S1-1 for diagram and timeline, and Figure S1-2 for transition intensity matrix governing possible model transitions). Starting from the study start time, *T_0_*, all patients began in the pre-trial health state. Each patient would accrue into the trial, i.e., move the enrolled health state, at an individual accrual time, *t_1k_,* based on a random draw from a uniform distribution (*T_0_, T_1max_*). From the enrolled health state, patients would transition to death based on the hazard rate, $\lambda_{12}$ drawn from an exponential distribution, at the patient’s event time, *t_2k_.* The R package *gems* was used to simulate the multistate data generation process.

*T_0_*

*t_1k_*

*t_2k_*

*T_1max_*

**Figure S1-1 Health states and timeline for simulation.**

$$TIM=\Lambda=\left[ \begin{matrix} -\lambda_{01} & \lambda_{01} & 0 \\ 0 & {-\lambda}_{12} & \lambda_{12} \\ 0 & 0 & 0 \end{matrix} \right]$$

**Figure S1-2 Transition intensity matrix for the simulation**

Individual event times for population were produced for each transition. The Tstop time in the row with trans = 1 represents *t_1k_*, and the Tstop time in the row for trans = 2 represents *t_2k_*, measured from *T_0_*. The time value represents the true event time from accrual, *t_2k_*, - *t_1k_*.


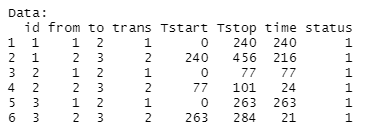

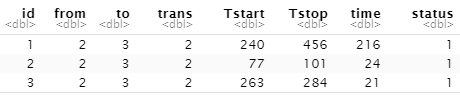


To simulate data resembling a clinical trial dataset, enrollment and death times were rounded to the nearest day, with the former rounded down and latter rounded up to ensure all event times > 0. Only data for the second transition (enrolled to death) were kept for analysis, which includes enrollment time (Tstart), event time from the start of the study (Tstop), and the difference, which represents event time from enrollment. When creating artificial datasets to analyze outcomes according to proportion of events observed, we artificially censor and therefore set the follow-up time from the study start time, *T_0_*, using event times from start of the study (Tstop) (see below). We then analyze survival for the cohort from enrollment. The purpose of random accrual times was to have similar effects as random censoring, such that follow-up time between individuals vary.

The simulation seed was set to 20. In each of the $i=1, 2, \ldots, n_{sim}=5,000$ repetitions, $sum(n_{obs})=1050$ patients were randomly sampled from the population of 50,000 individuals and allocated to create each of the six $n_{obs}$ sample size levels (i.e., first 30 patients are for sample size = 30, next 60 are for sample size = 60, etc). In the complete population data, all events are observed, so every patient has a status of 1.

In clinical trials where the time-to-event outcome is the primary outcome, a planned number of events is targeted to assess survival improvement, which informs the timing of the interim and final analyses. For each repetition and sample size, deciles of event times from the start of the study (study time) were estimated and used to create durations of follow-up times according to proportions of events experienced by the sample cohort. Then, artificially censored datasets were generated forming a stacked dataset with the individuals in each repetition and sample size repeated with event or censoring times from study enrollment and censoring status for each level of proportion of events, $p_{e}=\{0.1 ,0.2,\ldots,1.0\}$. As a result, we control how many patients in the artificially censored datasets have experienced the event, and vary it systematically by creating multiple, artificially censored datasets for each sample. This allowed us to evaluate the impact of changing the length of follow-up in that sample, in a manner similar to administratively censoring a clinical trial at a given time after targeted number of events are observed. The approach also allowed evaluation in a consistent manner across samples for different proportions, including complete follow-up (all events observed). For patients who have not enrolled by the study censor time corresponding to $p_{e}$, the patient event time was NA.

Time on trial, time2, and event status, status2, were determined as follows:

| Condition | Time on trial (time2) | Status on trial (status2) |
| --- | --- | --- |
| *t_stop_ < t_pe_* | *t_stop_ – t_start_* | 1 |
| *t_stop_ > t_pe_* | *t_pe_ - t_start_* | 0 (censored) |
| *t_start_ > t_pe_* | NA | NA |

For example, the times corresponding to each proportion of events, *t_pe_*, for the first sample size group of repetition 1 were:


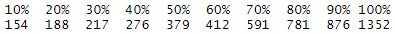


Time from the start of the study when 10% of events occurred was used to censor patients with longer observation times. By t = 154, 10% of events have occurred, so observation time is capped at 154 for the first level. The first patient has an event time in the observation window before *t_pe=0.1_* = 154, so their event was observed. The second patient had an event time longer than 154, so their observation was censored at t = 154 and status2 was censored. The third patient had not accrued to the trial by t = 154, thus their time on trial and status we NA.


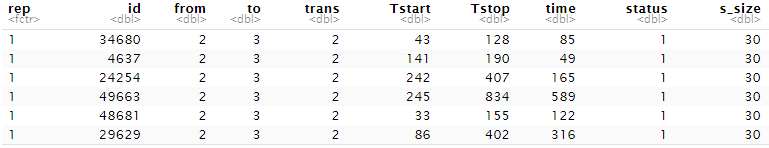

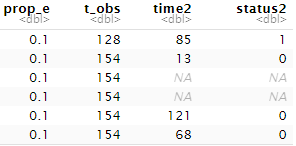


For $p_{e}$ = 0.2, the results were the same but with longer observation window for patients 2 and 6, and the event status =1 for patient 5.


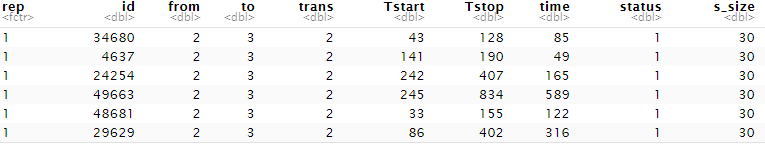

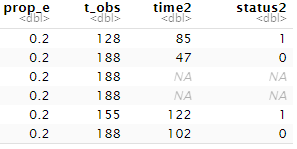


This approach was used to mimic how a clinical trial is conducted, in that patients accrue to the study over time from the study initiation date. As a result, when a study is stopped and administratively censored on a specific calendar day, patients have different lengths of follow-up from time of enrollment; thus, administrative censoring at specific times from study start is non-informative as it is unrelated to individual event times from start of enrollment.

## Formulas for performance measures

*Nonconverging or implausible fits*

The number of fitted models that failed to converge or met prespecified conditions that would render a fitted survival model as implausible were recorded and summarized.

*Proportion identifying the true distribution as best fitting*

The proportion of repetitions, $i=\{1,\ldots,n_{sim}\}$, for which the information criterion (IC) from an exponential survival model was lowest among the fitted distributions.

$$Best fitting=\frac{1}{n_{sim}}\sum_{i=1}^{n_{sim}} 1\left( IC_{exp,i}=\min\left( IC_{dist,i} \right) \right),$$

$$dist=\left\{ exponential,Weibull,lognormal,loglogistic,generalized Gamma,Gompertz \right\}$$

Distributions were fit using the R package *flexsurv* with default parameterization for these distributions.

Formulas for IC included:

$$AIC= -2 LL+2 p,$$

$$BIC= -2 LL+ p\log\left( n \right),$$

where $LL$ = log likelihood, $p$ = number of parameters, and $n$ = number of observations.

IC corrected for small samples (AICc and BICc) were also explored in the results, but not used for the full primary analysis given current guidance references the uncorrected AIC and BIC. The correction factor $n/(n-p-1)$was applied to the second term of IC formulas to derive AICc and BICc.

*Coverage and error*

The remaining population quantities of interest, $x$, included the median survival time, landmark one-year survival probability, 1% survival time, (${TH}_{pop})$, and population RMST estimated at ${TH}_{pop}$. The use of the 1% survival probability time was chosen as a practical modelling approach where a “lifetime” time horizon is defined to be the time point when less than 1% of patients are alive. Rate was not included as an estimand as a common parameter would not exist across distributions.

With respect to performance in targeting the population quantities of interest, $x$, the measures used are outlined below.

*Coverage:*

1. Coverage - the proportion of repetitions in the simulation whose confidence intervals contain the true population quantity.

$$Coverage=\frac{1}{n_{sim}}\sum_{i=1}^{n_{sim}} 1(\hat{x}_{low,i}<x<\hat{x}_{upp,i})$$

*Error:*

1. Mean absolute error (MAE) - the average absolute value of the distance between the estimate from each repetition, $\hat{x}_{i}$, and the true population fitted quantity, $x$.

$$MAE=\frac{1}{n_{sim}}\sum_{n=1}^{n_{sim}} |\hat{x}_{i}-x|$$

2. Mean absolute percentage error (MAPE) - the average absolute value of the distance between the estimate from each repetition, $\hat{x}_{i}$, and the true population fitted quantity, $x$, divided by the true population fitted quantity, $x.$


$$MAPE=\frac{1}{n_{sim}}\sum_{n=1}^{n_{sim}} \frac{|\hat{x}_{i}-x|}{x}$$

3. Root mean squared error (RMSE) - the root of the mean squared error between estimate from each repetition, $\hat{x}_{i}$, and the true population fitted quantity, $x$. Monte Carlo standard error was estimated using jackknife estimator (using R package *simhelpers*).

$$RMSE=\sqrt{\frac{1}{n_{sim}}\sum_{n=1}^{n_{sim}} (\hat{x}_{i}-x)^{2}}$$

4. Probability of 20% error - the proportion of repetitions that produced estimates >20% from the true value were summarized.

$$Probability of 20\% error= Pr\left( \frac{\left| \hat{x}_{i}-x \right|}{x}>20\% \right)$$

Though a minimally important difference for each estimand has not been established, a 20% difference was derived based on assessments from ASCO and ESMO committees that have defined minimal thresholds for clinical benefits of 20%, hazard ratio risk reductions of at least 0.8, and median improvements of 2.5-6 months (depending on clinical context) to be clinically meaningful in oncology [28,29].

Nonconverging or implausible fits, the proportion of repetitions identifying the true distribution as best-fitting, and the coverage and error (MAE, MAPE, RMSE, probability of 20% error) for each of the estimands were calculated for each combination of sample size and follow-up time. These performance measures were summarized along with respective Monte Carlo standard errors and plotted to illustrate the impact across sample sizes and degrees of censoring.
