# Supplementary material for: Impact of limited sample size and follow-up on single event survival extrapolation for health technology assessment: a simulation study
Source: BMC Med Res Methodol. 2021 Dec 18;21:282. doi: 10.1186/s12874-021-01468-7 (PMC8684239; doi:10.1186/s12874-021-01468-7)
Supplement: Supplementary file 2 — Additional file 2. Additional details for simulated populations and datasets. [file 12874_2021_1468_MOESM2_ESM.docx]

# Supplemental File 2: Additional details for simulated populations and datasets

Table of Contents

[Supplemental File 2: Additional details for simulated populations and datasets 1](#_Toc82033015)

[Scenario descriptions 2](#_Toc82033016)

[Population survival curves 3](#_Toc82033017)

[Population fitted values (estimands) 4](#_Toc82033018)

[Proportion of samples accrued by each level of proportion of events 5](#_Toc82033019)

[Number of nonconverging or implausible fits across all four scenarios 6](#_Toc82033020)

[Proportion identifying the true distribution as best-fitting across all four scenarios 7](#_Toc82033021)

## Scenario descriptions

Four scenarios were conducted to examine two levels of accrual and event rates, made up of a combination of short (approximately 9 months maximum accrual time, 9 months median event time) or long times (approximately 30 months maximum accrual time, 30 months median event time) (Table S2-1). The accrual time range was chosen after reviewing published statistical analysis plans in oncology trials, including lung, breast, and hematologic cancers. The factorial design permitted an assessment of any relationship between accrual time relative to event time. Each scenario was conducted with $n_{sim}$= 5,000 repetitions.

Table S2-1. Scenario descriptions

| Scenarios | | Accrual time | |
| --- | --- | --- | --- |
|  |  | Short | Long |
| Event time | Short (high rate) | 1 (Base)-High event rate, short accrual | 2-High event rate, long accrual |
|  | Long (low rate) | 3-Low event rate, short accrual | 4-Low event rate, long accrual |

## Population survival curves


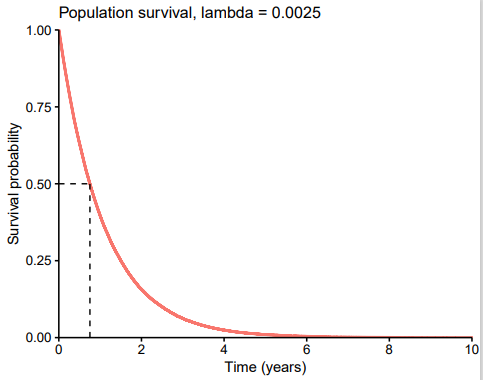

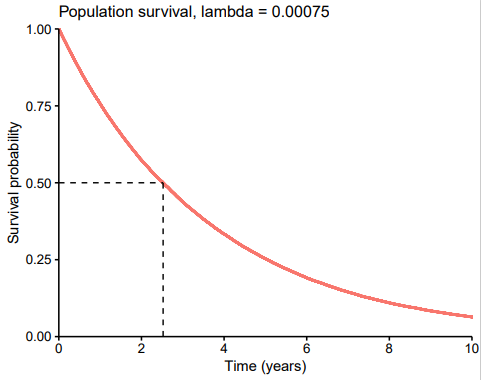


**Figure S2-1 Population survival curves for lambda = 0.0025 (scenarios 1 and 2) and for lambda = 0.00075 (scenarios 3 and 4).**

## Population fitted values (estimands)

Table S2-2. Population fitted values for each scenario (times measured in days)

|  | Scenario 1 | Scenario 2 | Scenario 3 | Scenario 4 |
| --- | --- | --- | --- | --- |
| Rate | 0.00253 (0.00251 – 0.00255) | 0.00249 (0.0247 – 0.251) | 0.000745 (0.000739 – 0.000752) | 0.000754 (0.000747 – 0.000760) |
| Max accrual time | 9 months | 30 months | 9 months | 30 months |
| OS Median | 274 (272 – 277) | 278 (276 – 281) | 930 (922 – 938) | 920 (912 – 928) |
| One-year survival | 39.7% (39.4% – 40.0%) | 40.3% (39.4% – 40.6%) | 76.2% (76.0% – 76.4%) | 75.9% (75.8% – 76.1%) |
| Time horizon, *TH_pop_* | 1822 (1806 – 1837) | 1849 (1832 – 1866) | 6177 (6123 – 6236) | 6112 (6057 – 6169) |
| RMST at *TH_pop_* | 392 (388 – 395) | 397 (394 – 400) | 1328 (1316 – 1339) | 1315 (1304 – 1326) |
| Max event time | 3,808 | 4,946 | 15,685 | 13,129 |

## Proportion of samples accrued by each level of proportion of events

As patients accrued to study cohorts over time, analysis after a small proportion of events can be conducted before the full target population has accrued. When accrual time and event times were similar (scenarios 1 and 4), on average about 30% of events had been observed by the time trials regularly had accrued the full target sample size. In circumstances where trial design dictates that data not be analyzed until a minimum time after the full target sample size had accrued, there would often be a reasonable proportion of events already observed; the exception of which being when the event time is long (i.e., event rate is very low) in relation to accrual time (scenario 3).


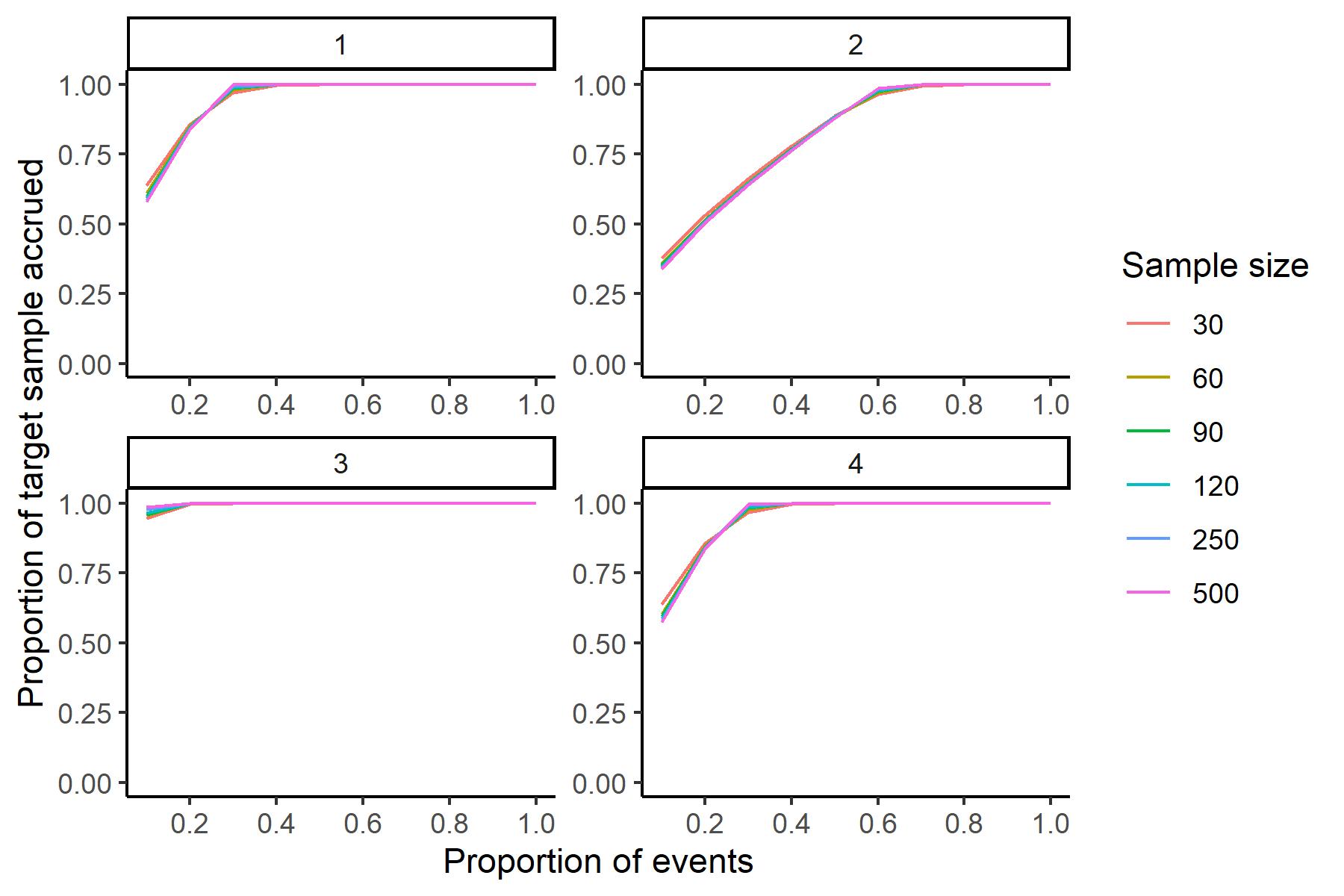


Figure S2-2 Proportion of target sample size accrued at each level of proportion of events for each scenario

Scenarios: 1- high event rate, short accrual

2- high event rate, long accrual

3- low event rate, short accrual

4- low event rate, long accrual

## Number of nonconverging or implausible fits across all four scenarios


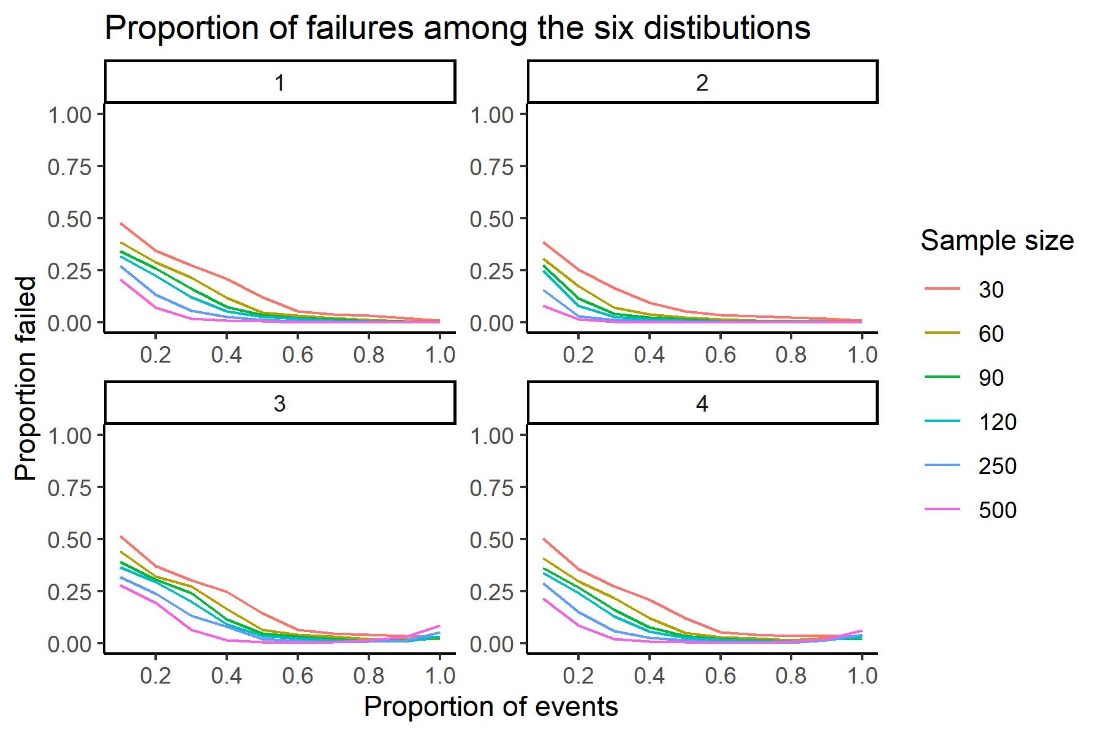


**Figure** **S2-3 Proportion of fitted models (across 5,000 repetitions and 6 distributions for each) that failed to converge or produced implausible results for each sample size and level of proportion of events**

Scenarios: 1- high event rate, short accrual

2- high event rate, long accrual

3- low event rate, short accrual

4- low event rate, long accrual

## Proportion identifying the true distribution as best-fitting across all four scenarios

**
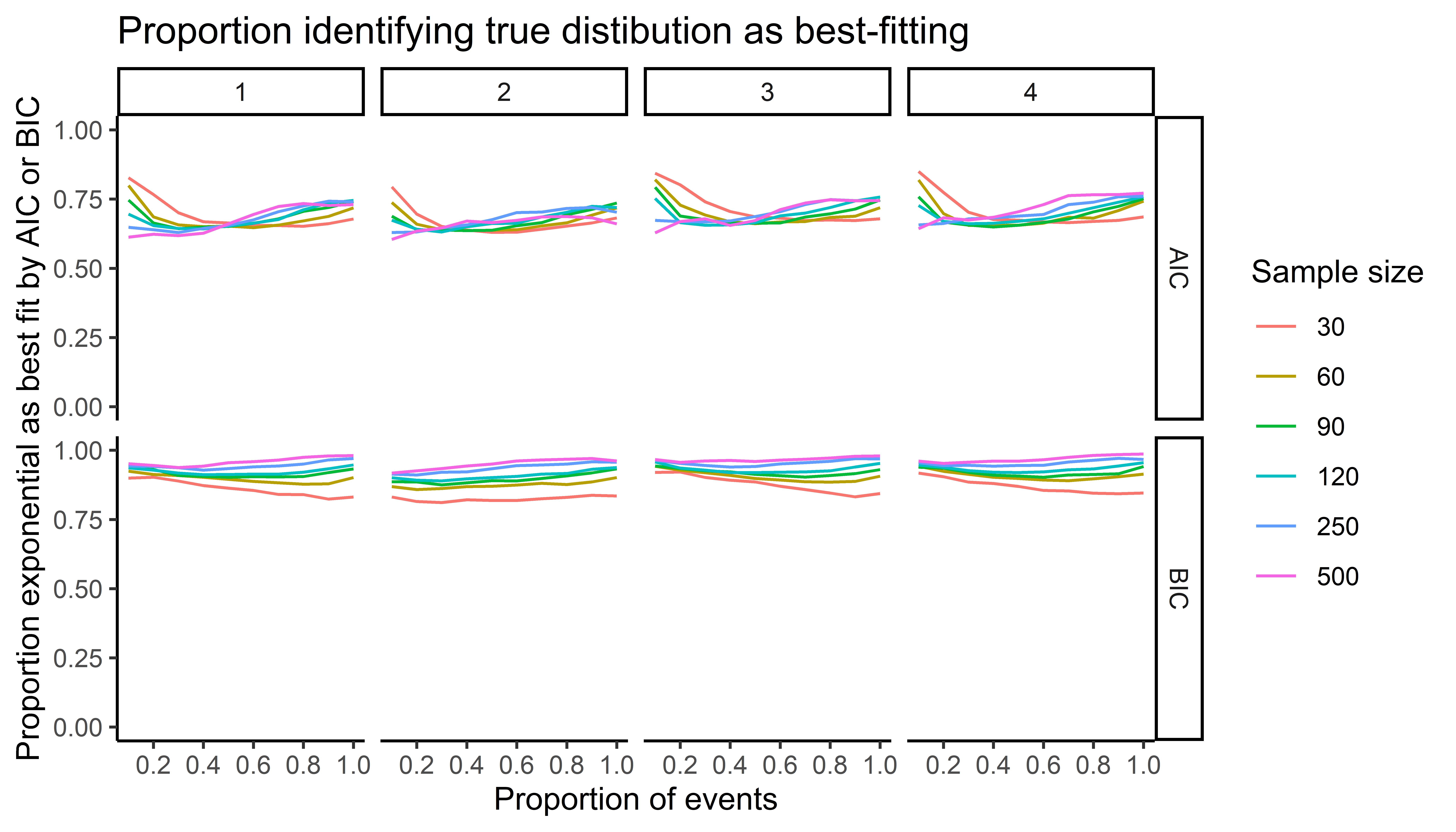
**

Figure S2-4 Proportion of repetitions in the simulation that identified the best-fitting distribution to be the true exponential distribution, according to AIC or BIC in each scenario

Scenarios: 1- high event rate, short accrual

2- high event rate, long accrual

3- low event rate, short accrual

4- low event rate, long accrual
