# Supplementary material for: Impact of limited sample size and follow-up on single event survival extrapolation for health technology assessment: a simulation study
Source: BMC Med Res Methodol. 2021 Dec 18;21:282. doi: 10.1186/s12874-021-01468-7 (PMC8684239; doi:10.1186/s12874-021-01468-7)
Supplement: Supplementary file 3 — Additional file 3. Additional results for Scenario 1. [file 12874_2021_1468_MOESM3_ESM.docx]

# Supplemental File 3: Additional results for Scenario 1

Table of Contents

[Supplemental File 3: Additional results for Scenario 1 1](#_Toc82024660)

[Coverage comparing uncorrected and corrected IC 2](#_Toc82024661)

[Mean Absolute Error (MAE) 3](#_Toc82024662)

[Root Mean Squared Error (RMSE) 4](#_Toc82024663)

[Mean Absolute Percentage Error (MAPE) comparing uncorrected and corrected IC 5](#_Toc82024664)

## Coverage comparing uncorrected and corrected IC

Coverage results comparing uncorrected and corrected information criteria (IC) , showing little improvement in results from corrected IC


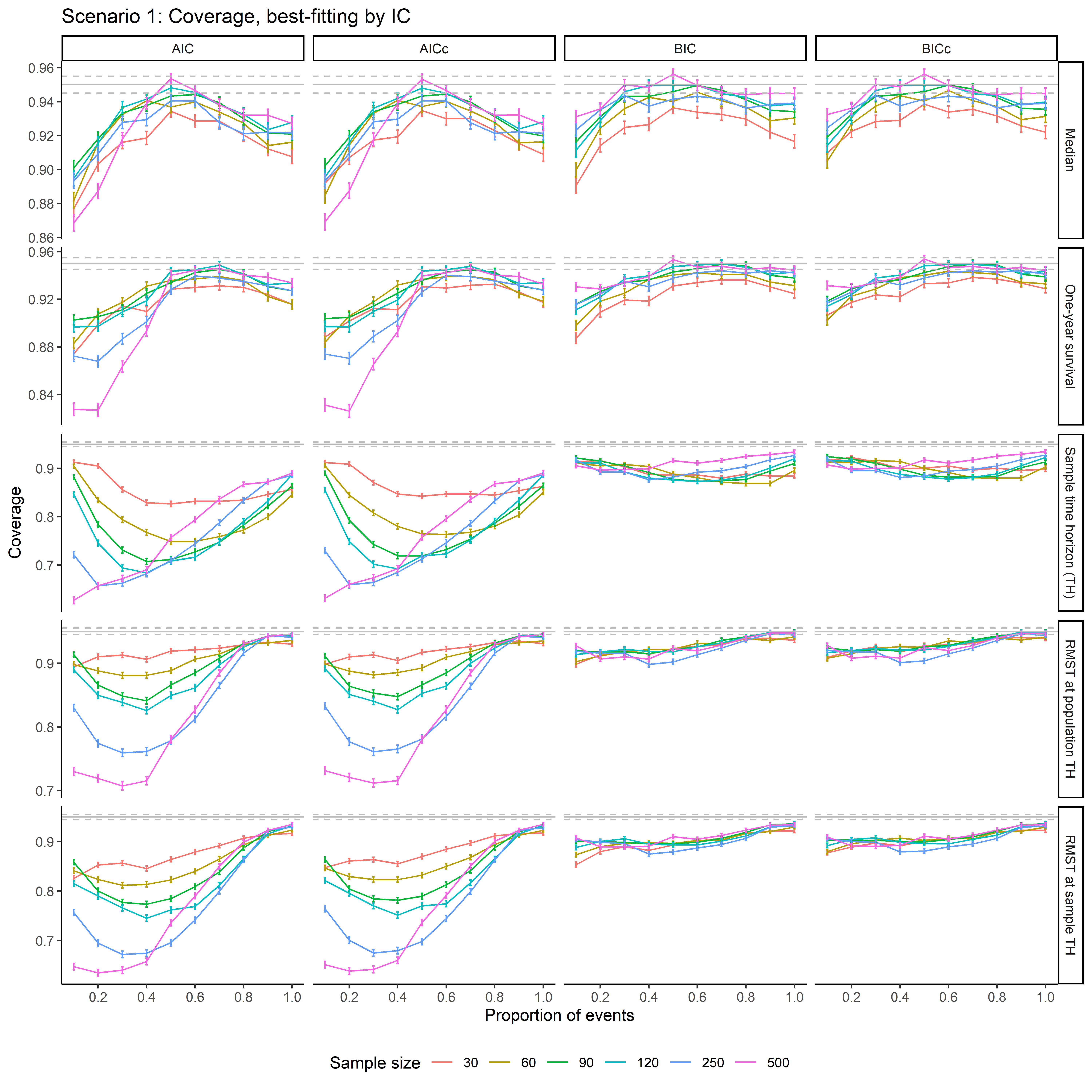


Figure S3-1 Coverage across proportions of events and sample sizes when distribution is chosen based on corrected or uncorrected AIC or BIC (scenario 1)

## Mean Absolute Error (MAE)

Additional error measures – Mean absolute error (MAE) show similar patterns in results and implications to the main analysis with MAPE.

**
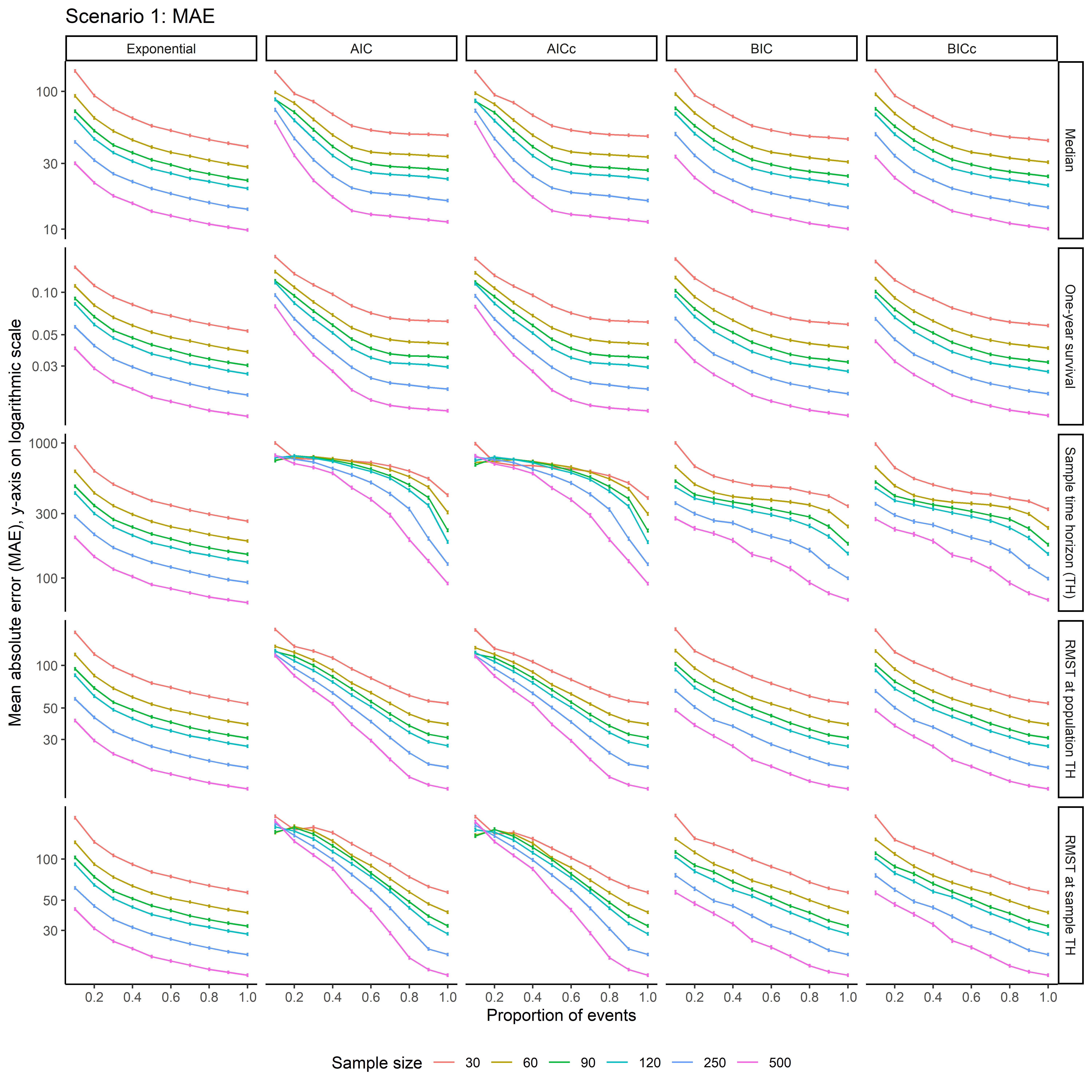
 Figure** **S3-2 Mean absolute error (MAE) (y-axis on logarithmic scale) across proportions of events and sample sizes** **when distribution is correctly specified as exponential or chosen based on information criteria (IC) (scenario 1)**

## Root Mean Squared Error (RMSE)

Additional error measures – root mean square error (RMSE) show similar patterns in results and implications to the main analysis with MAPE. Similar patterns also with MAE, with slightly larger magnitudes, reflecting more weight from estimates with large deviations.

**
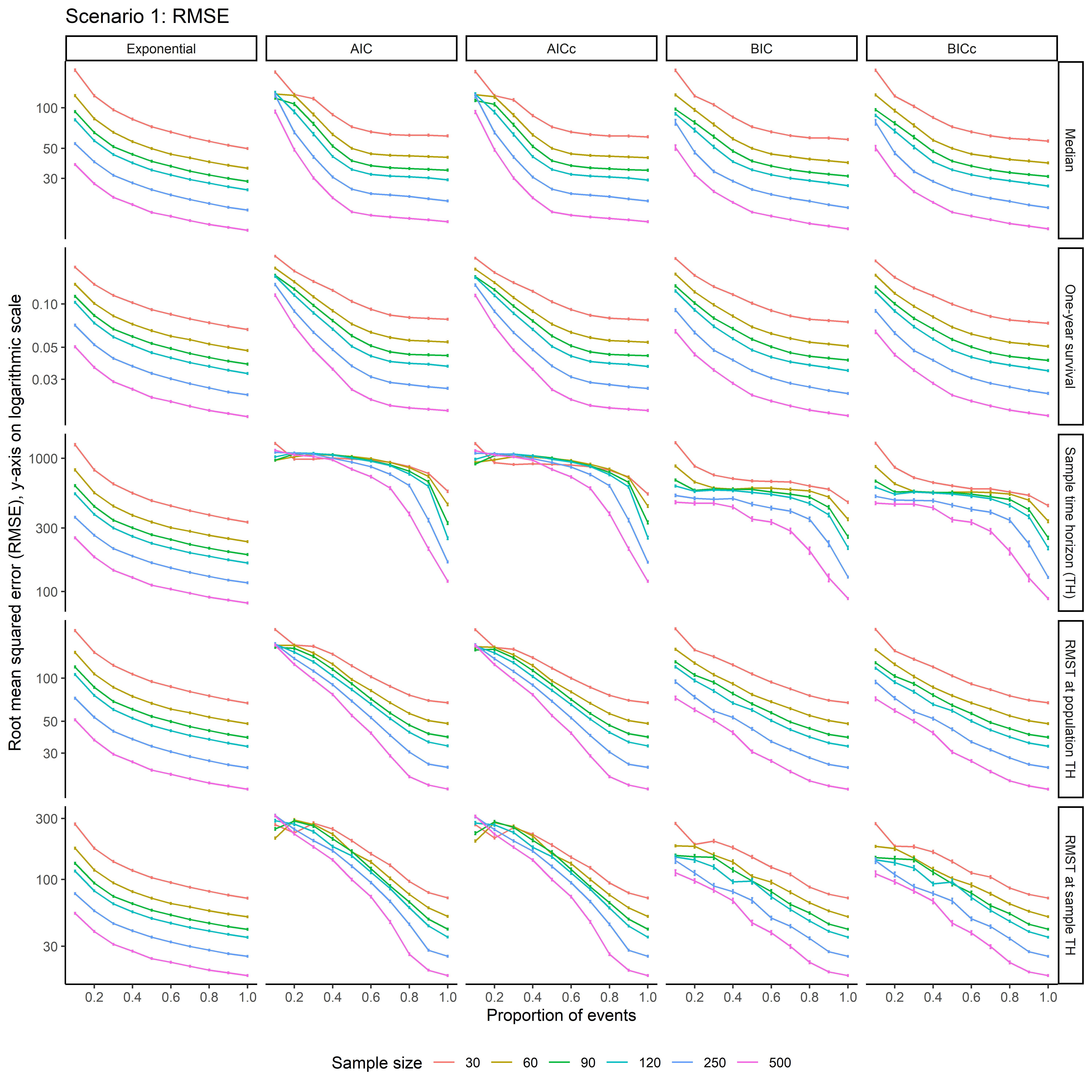
**

**Figure** **S3-3 Root mean squared error (RMSE) (y-axis on logarithmic scale) across proportions of events and sample sizes** **when distribution is correctly specified as exponential or chosen based on information criteria (IC) (scenario 1)**

## Mean Absolute Percentage Error (MAPE) comparing uncorrected and corrected IC

Mean absolute percentage error (MAPE) results comparing uncorrected or corrected information criteria (IC), showing little improvement in results from corrected IC

**
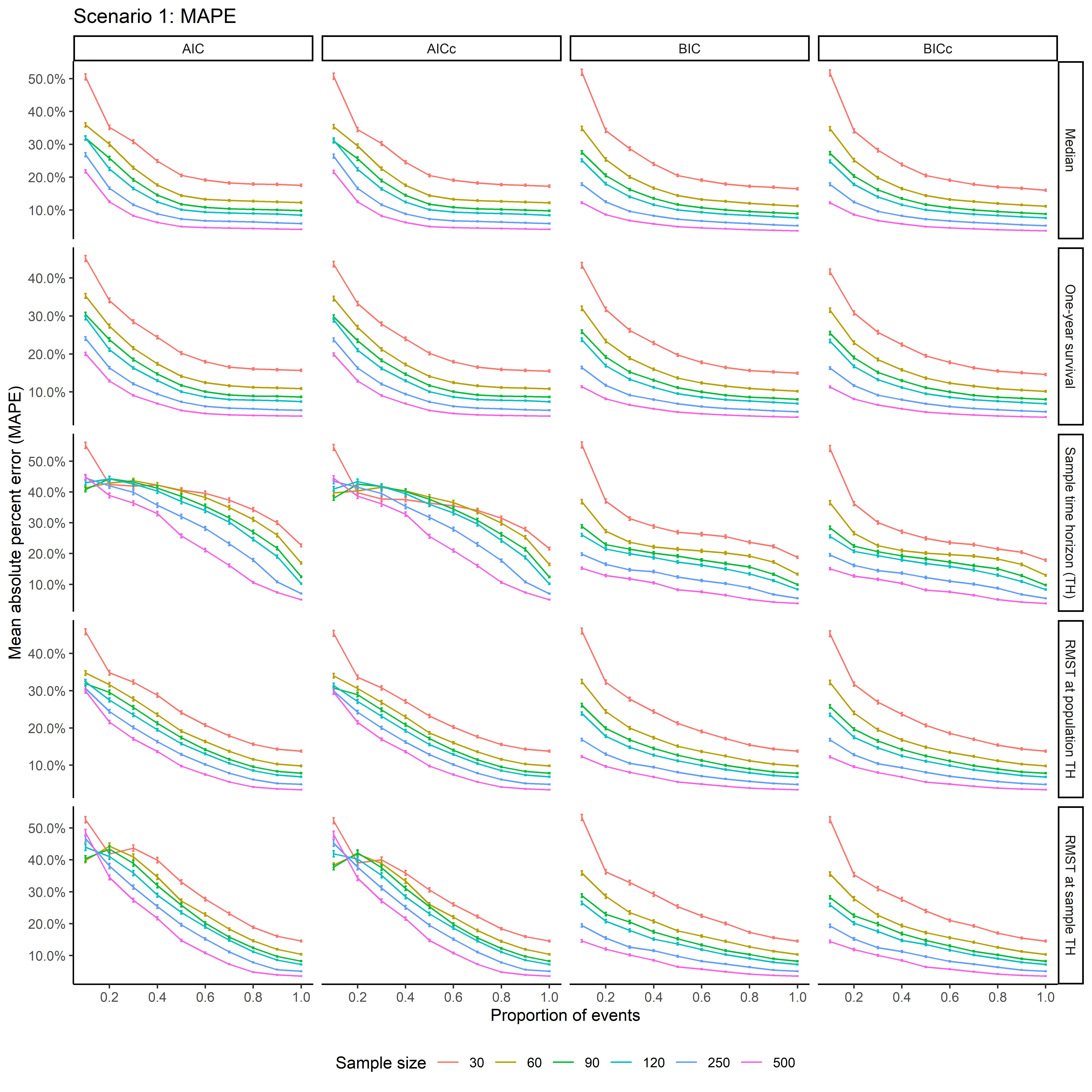
**

**Figure** **S3-4 Mean absolute percentage error (MAPE)** **across proportions of events and sample sizes when distribution is chosen based on corrected or uncorrected AIC or BIC (scenario 1)**
