# Supplementary material for: Impact of limited sample size and follow-up on single event survival extrapolation for health technology assessment: a simulation study
Source: BMC Med Res Methodol. 2021 Dec 18;21:282. doi: 10.1186/s12874-021-01468-7 (PMC8684239; doi:10.1186/s12874-021-01468-7)
Supplement: Supplementary file 4 — Additional file 4. Additional results comapring across all four scenarios. [file 12874_2021_1468_MOESM4_ESM.docx]

## Supplemental File 4: Additional results comparing across all four scenarios

Table of Contents

[Supplemental File 4: Additional results comparing across all four scenarios 1](#_Toc82029283)

[Coverage 2](#_Toc82029284)

[Mean absolute percentage error (MAPE) 5](#_Toc82029285)

[Probability of >20% difference 8](#_Toc82029286)

The contents of this Supplemental File can be accessed in an interactive tool available at: <https://survsim.shinyapps.io/survsim>

## Coverage


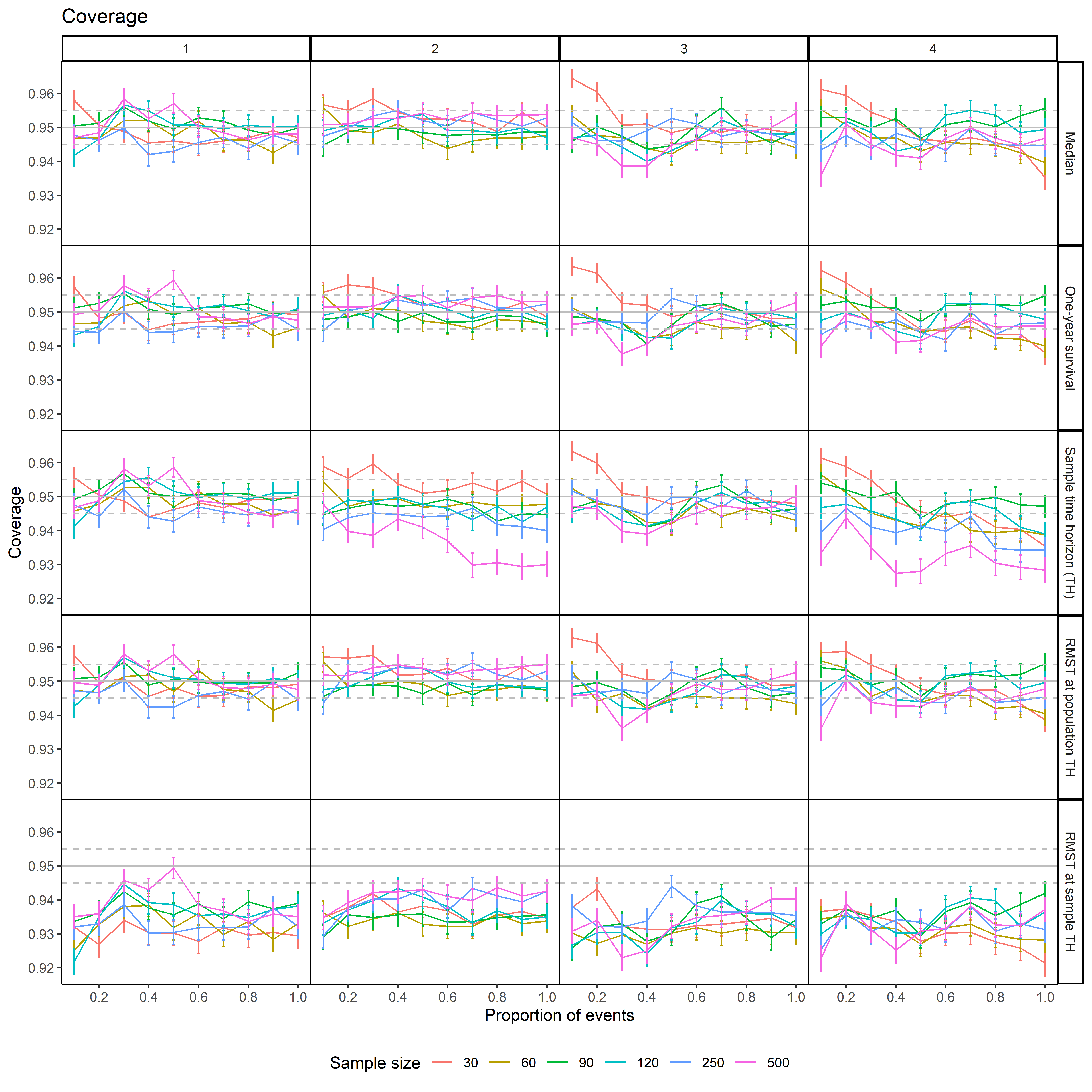
Figure S4-1 Coverage at each sample size and level of proportion of events across scenarios when distribution correctly specified as exponential

Scenarios: 1- high event rate, short accrual

2- high event rate, long accrual

3- low event rate, short accrual

4- low event rate, long accrual


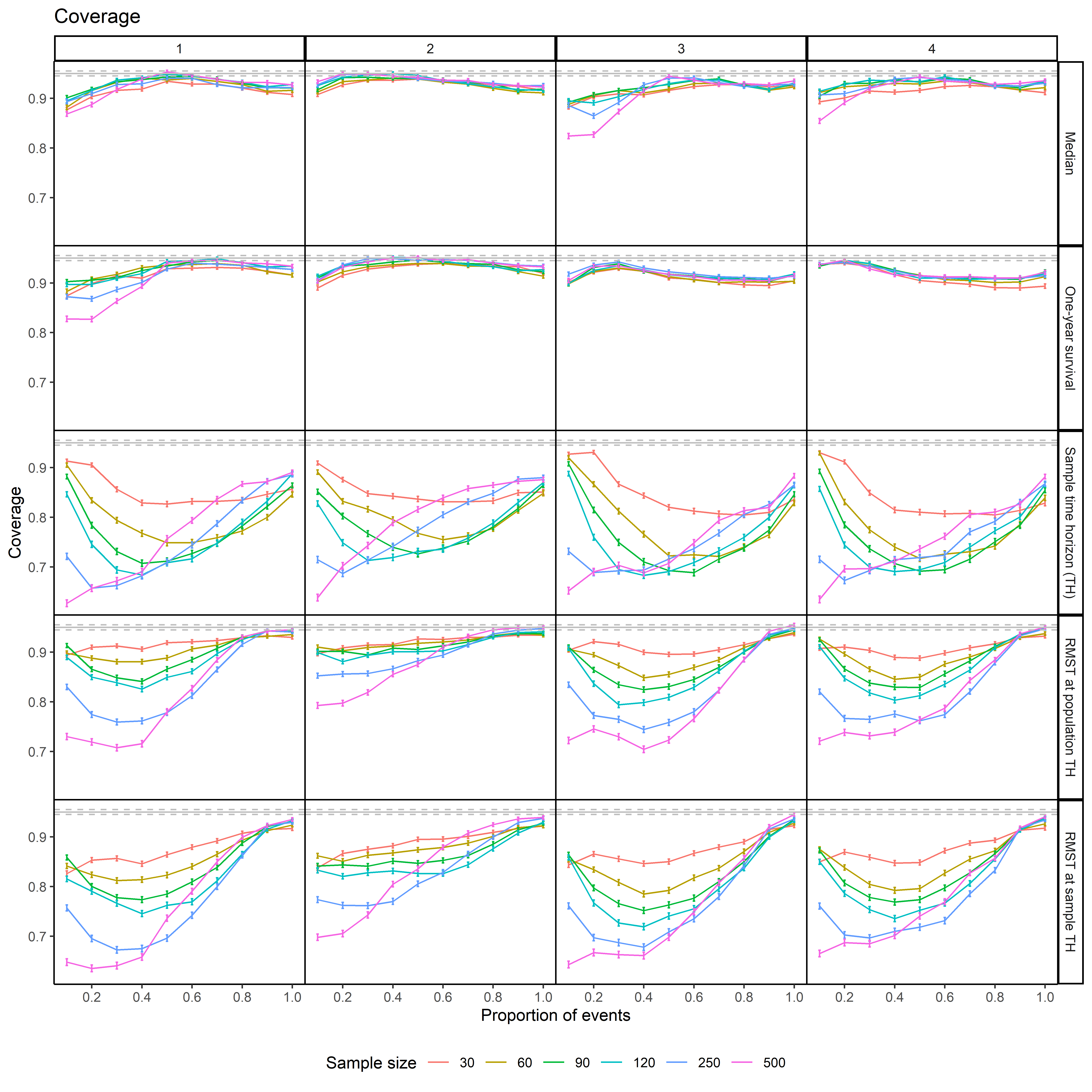


**Figure** **S4-2 Coverage at each sample size and level of proportion of events across scenarios when distribution was chosen by AIC**

Scenarios: 1- high event rate, short accrual

2- high event rate, long accrual

3- low event rate, short accrual

4- low event rate, long accrual

**
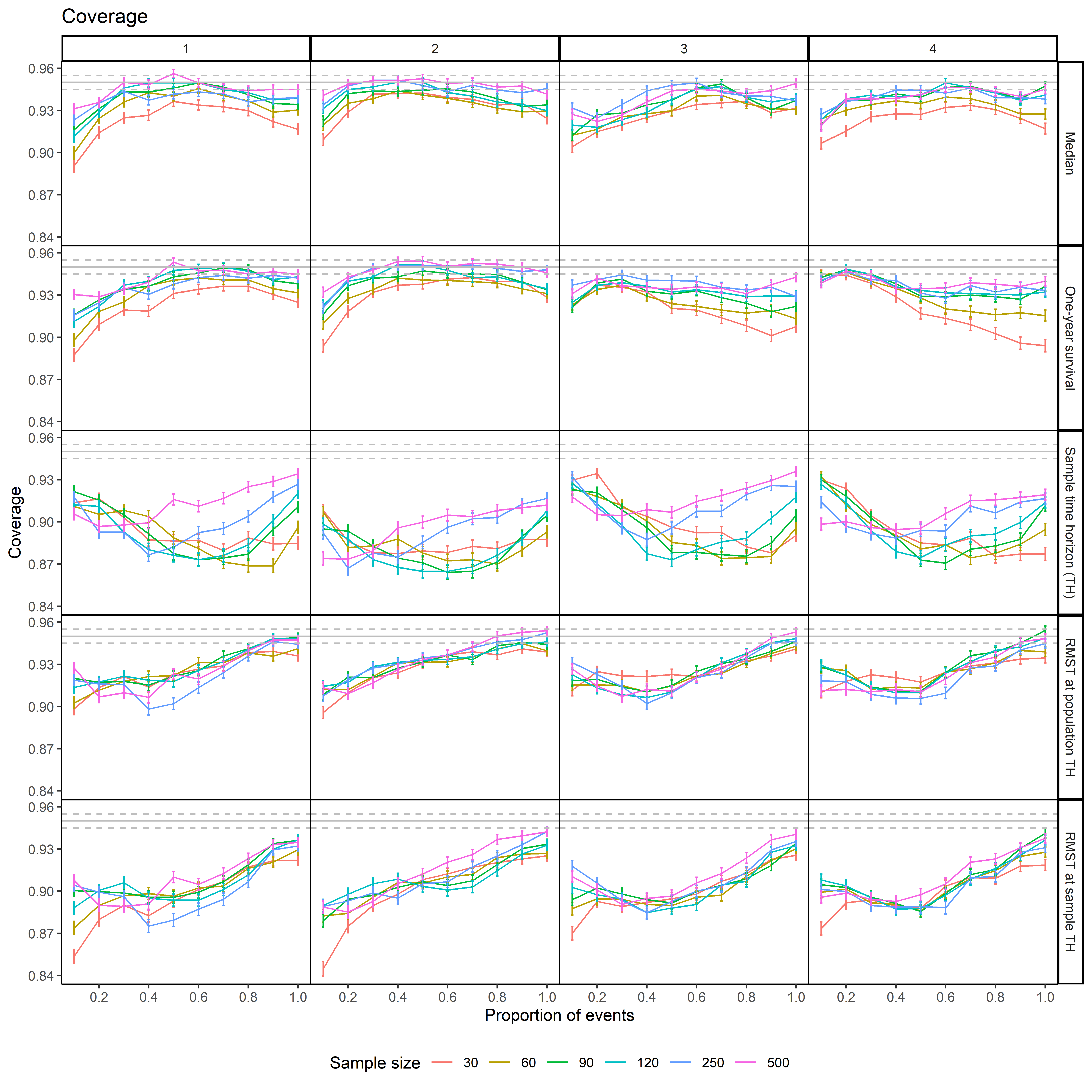
**

**Figure** **S4-3 Coverage at each sample size and level of proportion of events for each scenario when distribution was chosen by BIC**

Scenarios: 1- high event rate, short accrual

2- high event rate, long accrual

3- low event rate, short accrual

4- low event rate, long accrual

## Mean absolute percentage error (MAPE)


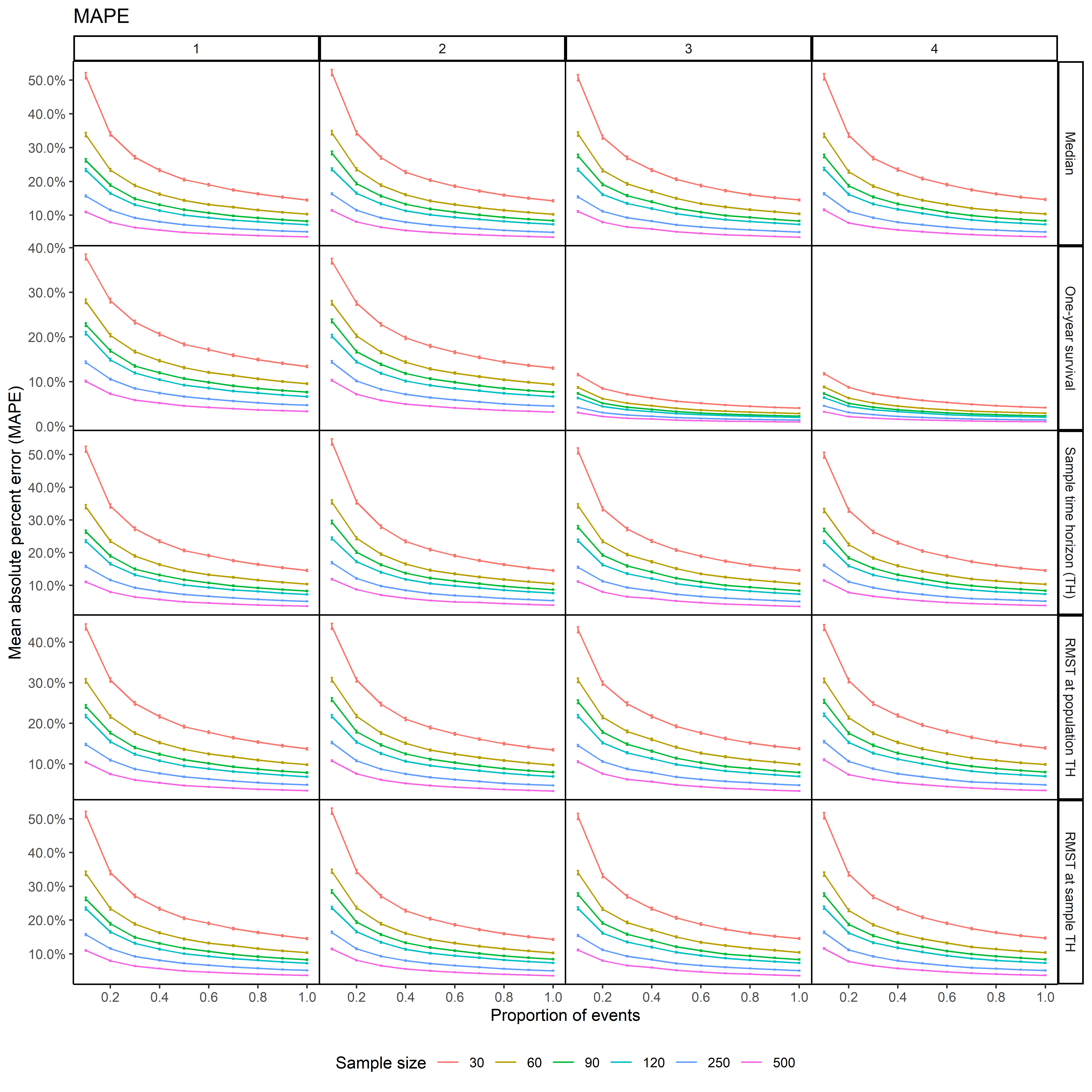
 Figure S4-4 Mean absolute percentage error (MAPE) at each sample size and level of proportion of events for each scenario when distribution correctly specified as exponential

Scenarios: 1- high event rate, short accrual

2- high event rate, long accrual

3- low event rate, short accrual

4- low event rate, long accrual

**
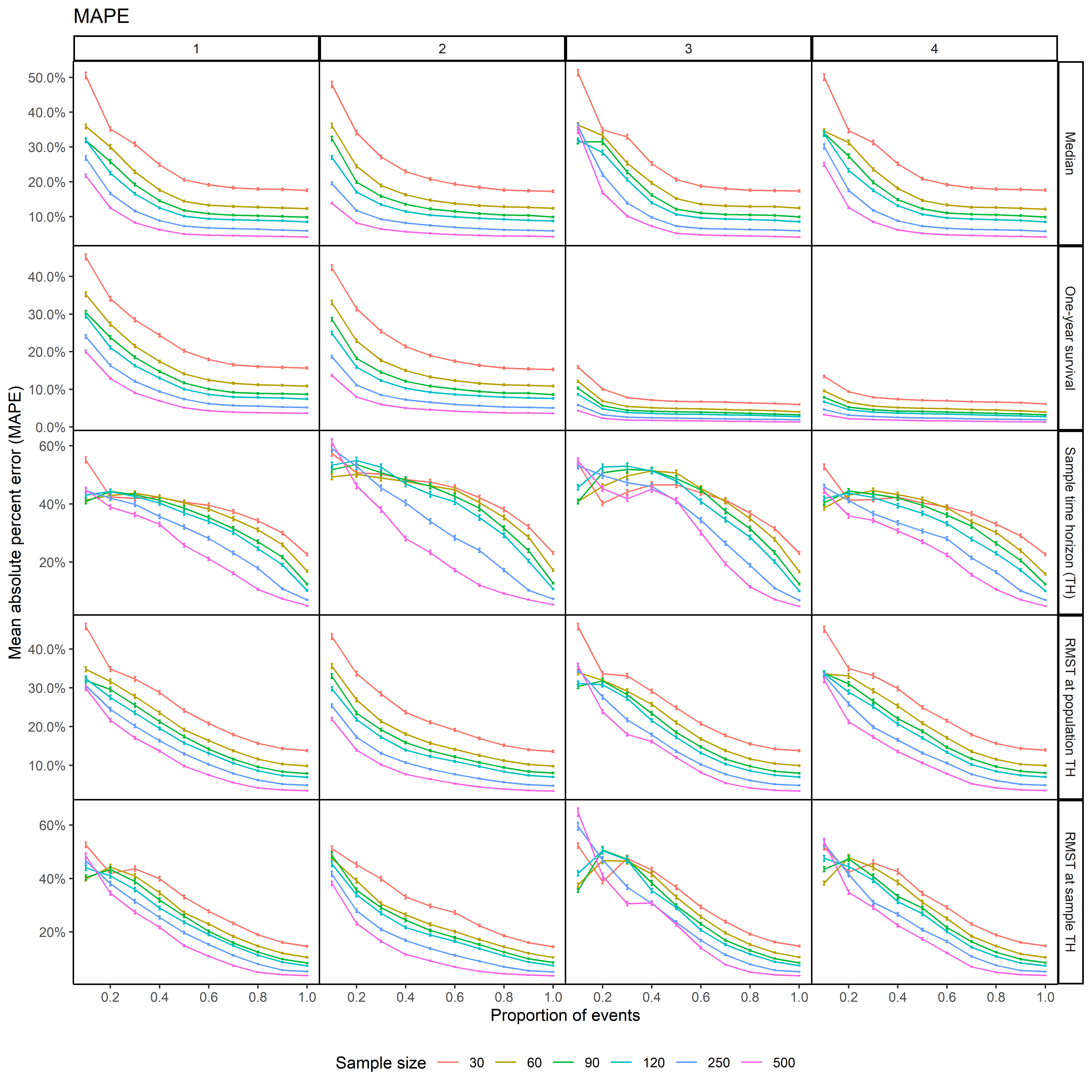
**

**Figure** **S4-5 Mean absolute percentage error (MAPE) at each sample size and level of proportion of events for each scenario when distribution is chosen by AIC**

Scenarios: 1- high event rate, short accrual

2- high event rate, long accrual

3- low event rate, short accrual

4- low event rate, long accrual

**
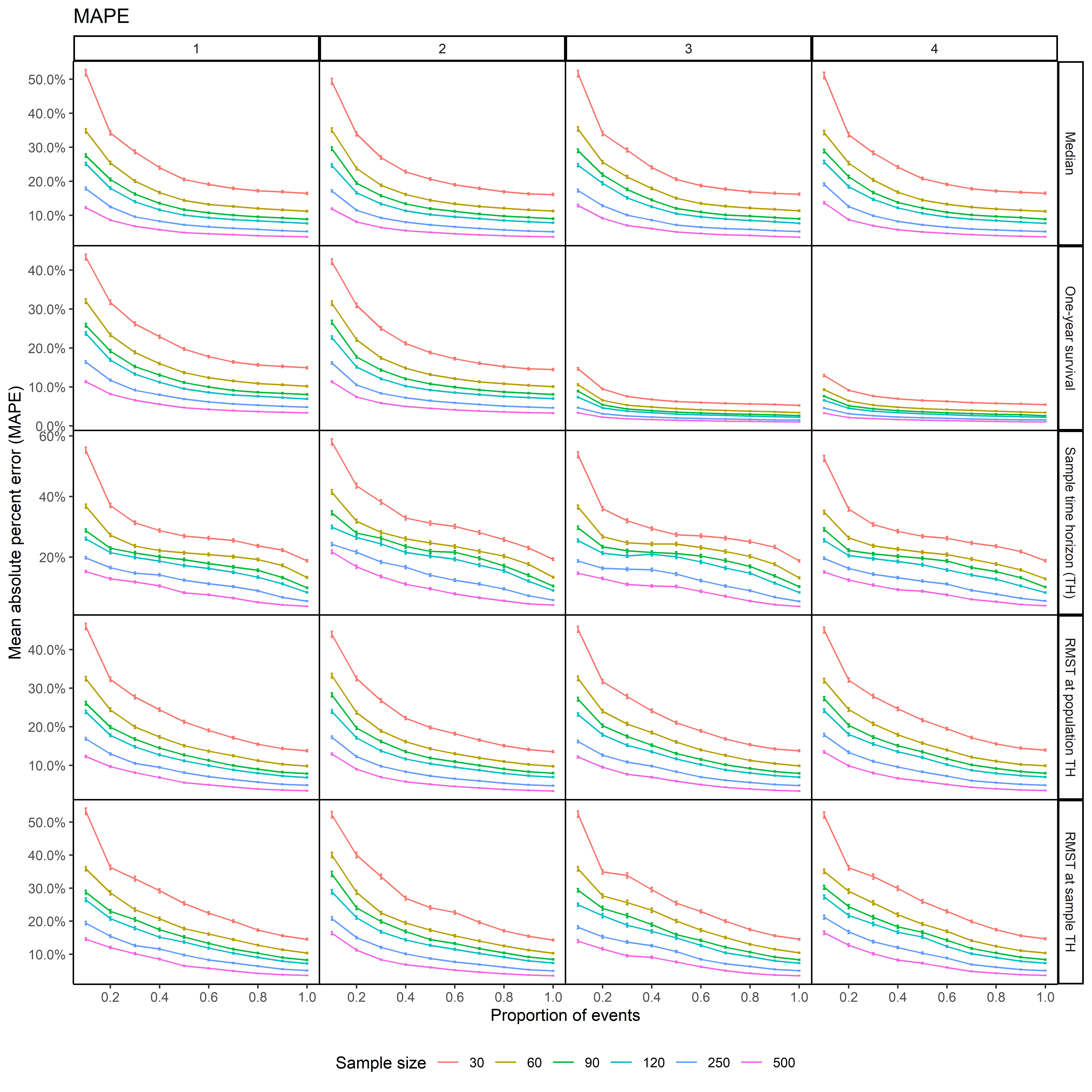
**

**Figure S4-6 Mean absolute error (MAPE) at each sample size and level of proportion of events for each scenario when distribution is chosen by BIC**

Scenarios: 1- high event rate, short accrual

2- high event rate, long accrual

3- low event rate, short accrual

4- low event rate, long accrual

## Probability of >20% difference


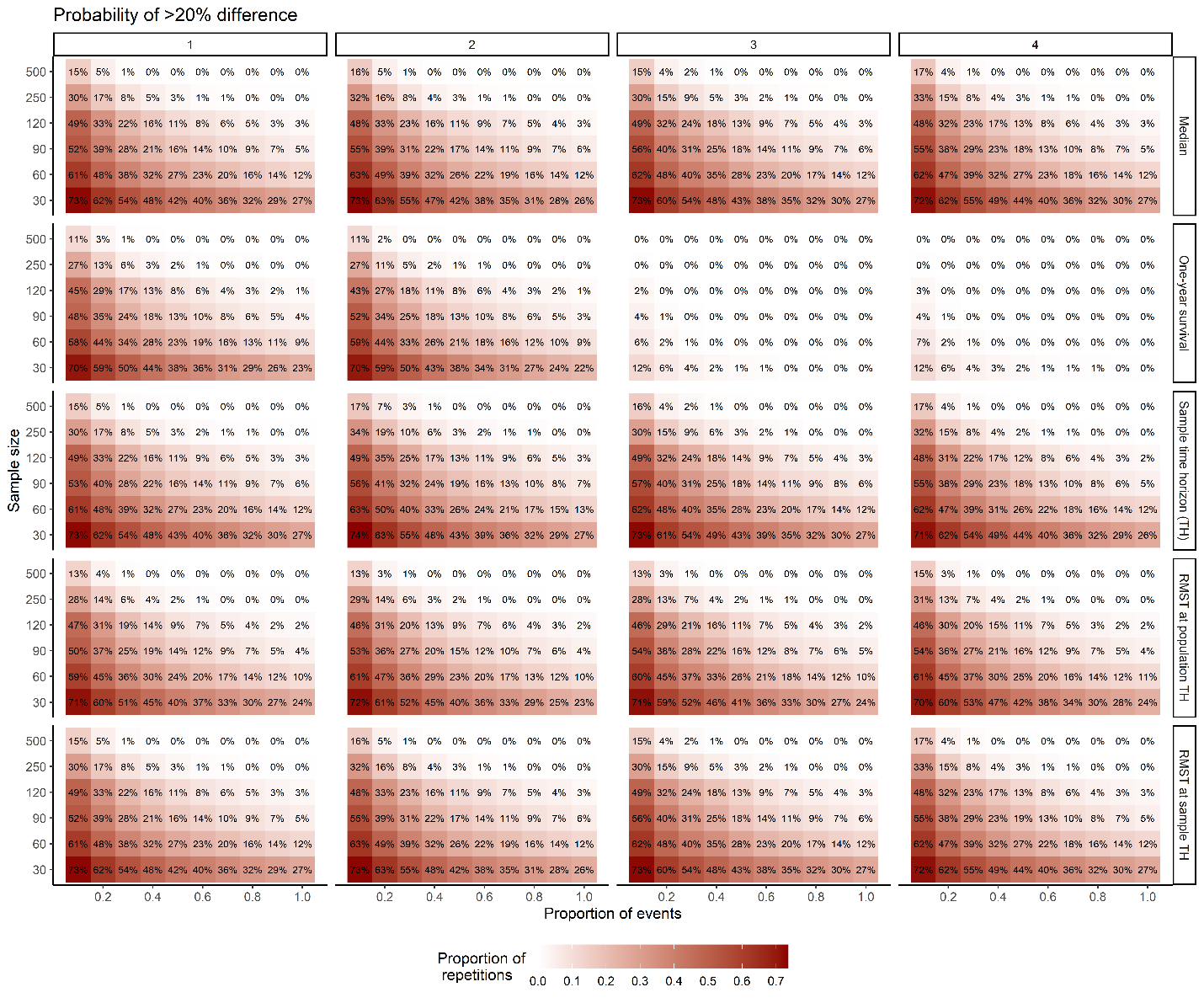


**Figure** **S4-7 Proportion of repetitions with estimates > 20% different from population fitted value across scenarios with distribution correctly specified as exponential**

Scenarios: 1- high event rate, short accrual

2- high event rate, long accrual

3- low event rate, short accrual

4- low event rate, long accrual

| **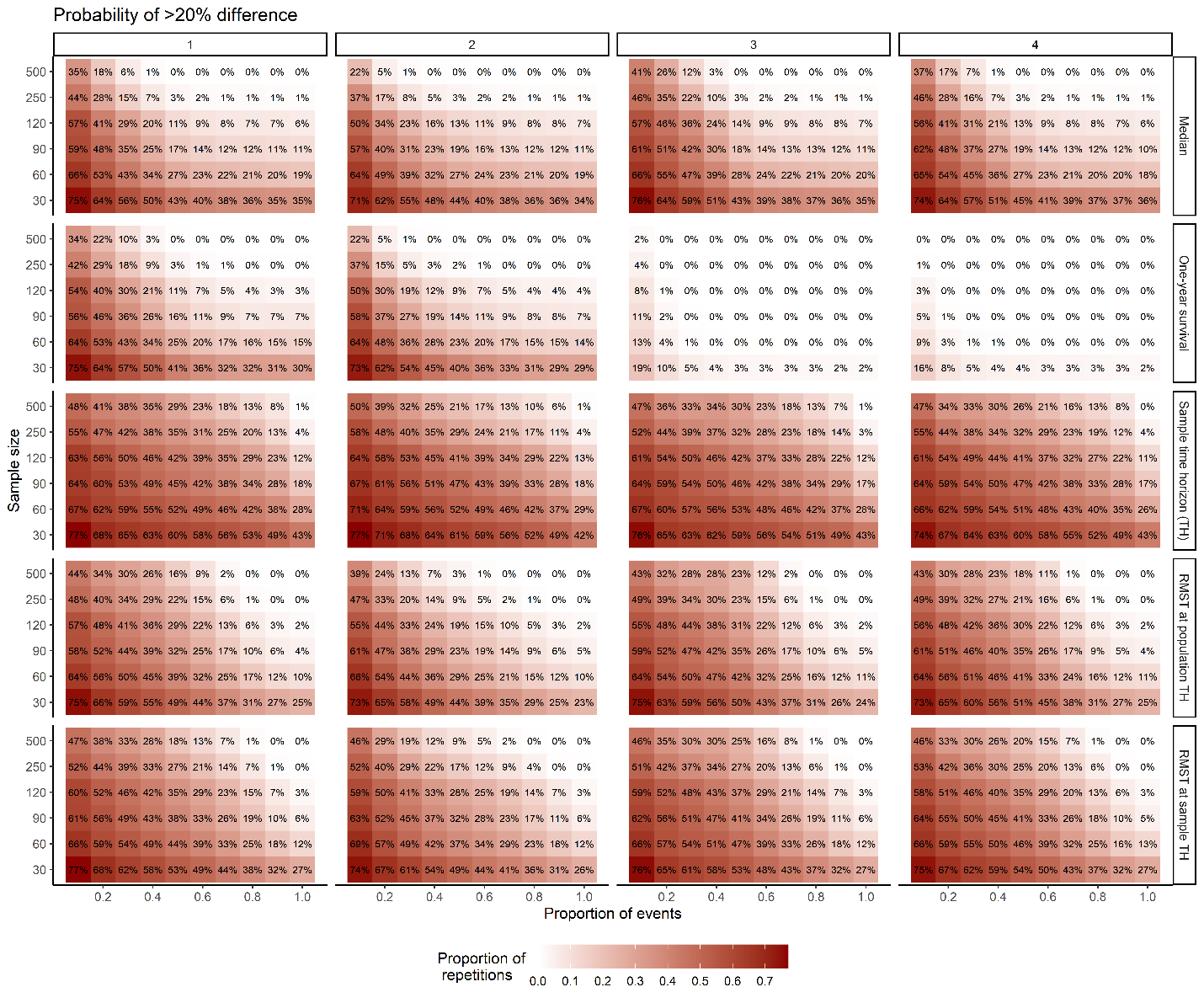**  **Figure S4-8 Proportion of repetitions with estimates > 20% different from population fitted value across scenarios with distribution specified by AIC**  Scenarios: 1- high event rate, short accrual  2- high event rate, long accrual  3- low event rate, short accrual  4- low event rate, long accrual  **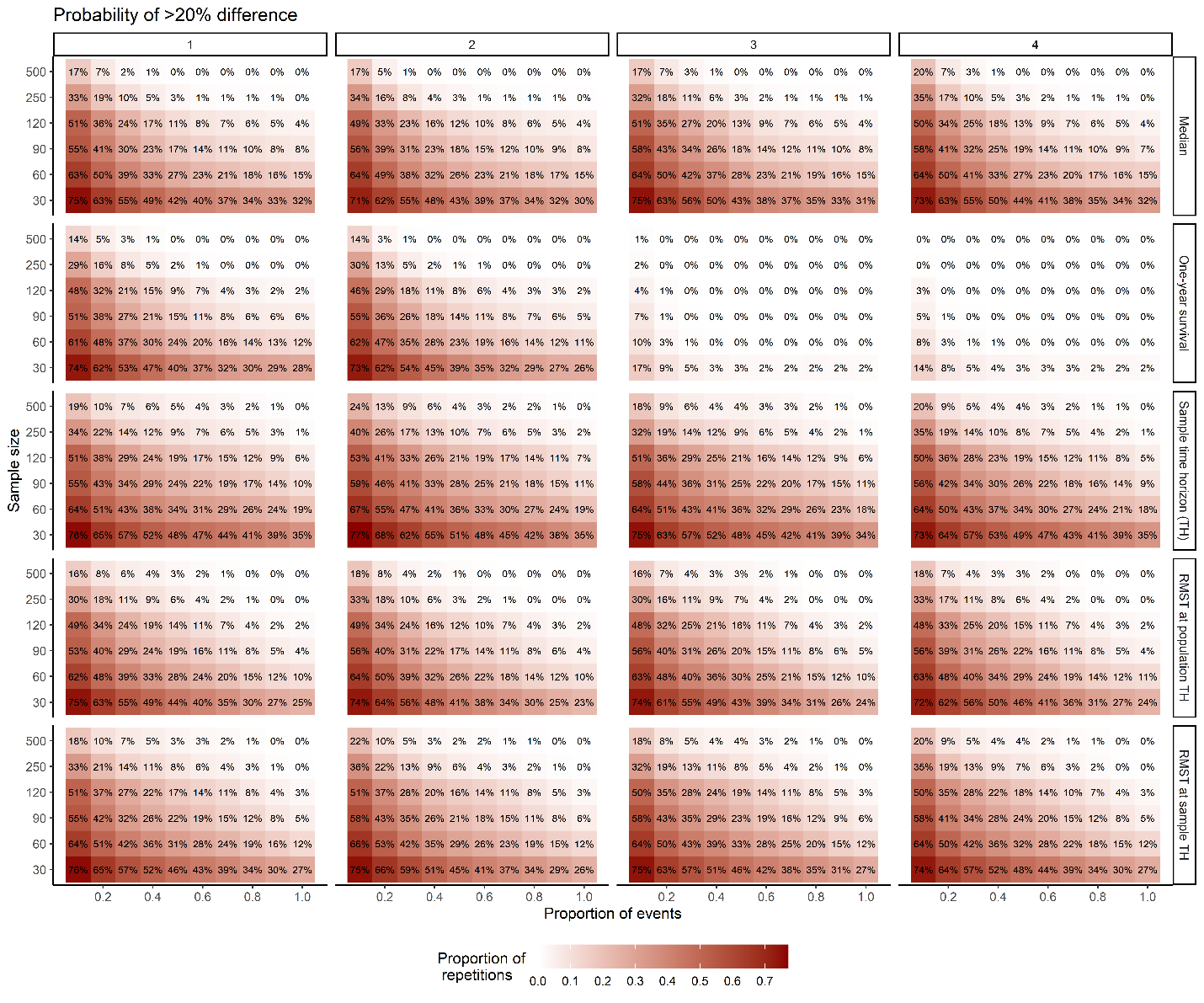**  **Figure** **S4-9 Proportion of repetitions with estimates > 20% different from population fitted value across scenarios with distribution specified by BIC**  Scenarios: 1- high event rate, short accrual  2- high event rate, long accrual  3- low event rate, short accrual  4- low event rate, long accrual |
| --- |
